# Estimation of substitution and indel rates via k-mer statistics

**DOI:** 10.1101/2025.05.14.653858

**Published:** 2025-05-18

**Authors:** Mahmudur Rahman Hera, Paul Medvedev, David Koslicki, Antonio Blanca

**Affiliations:** School of Electrical Engineering and Computer Science, Pennsylvania State University, USA; Department of Computer Science and Engineering, The Pennsylvania State University, University Park, USA; Department of Biochemistry and Molecular Biology, The Pennsylvania State University, University Park, USA; Huck Institutes of the Life Sciences, The Pennsylvania State University, University Park, USA; School of Electrical Engineering and Computer Science, Pennsylvania State University, USA; Department of Biology, Pennsylvania State University, USA; Huck Institutes of the Life Sciences, Pennsylvania State University, USA; School of Electrical Engineering and Computer Science, Pennsylvania State University, USA

**Keywords:** k-mer, mutation rate, indel, alignment-free, estimation, substitution, insertion, deletion, Applied computing → Computational biology, Theory of computation → Theory and algorithms for application domains, Mathematics of computing → Probabilistic inference problems

## Abstract

Methods utilizing k-mers are widely used in bioinformatics, yet our understanding of their statistical properties under realistic mutation models remains incomplete. Previously, substitution-only mutation models have been considered to derive precise expectations and variances for mutated k-mers and intervals of mutated and nonmutated sequences. In this work, we consider a mutation model that uses insertions and deletions in addition to single-nucleotide substitutions. Within this framework, we derive closed-form k-mer-based-estimators for the three fundamental mutation parameters: substitution rate, deletion rate, and average insertion length. We provide statistics of k-mers under this model and theoretical guarantees via concentration inequalities, ensuring correctness under reasonable conditions. Empirical evaluations on simulated evolution of genomic sequences confirm our theoretical findings, demonstrating that accounting for indel signals allows for accurate estimation of mutation rates and improves upon the results obtained by considering a substitution-only model. An implementation of estimating the mutation parameters from a pair of FASTA files is available here: github.com/mahmudhera/estimate_rates_using_mutation_model.git. The results presented in this manuscript can be reproduced using the code available here: github.com/mahmudhera/est_rates_experiments.git.

## 1 Introduction

Estimating the mutation rate between two evolutionarily related sequences is a classical question in molecular evolution, with roots that pre-date the genomics era [21]. Early quantitative efforts focused on amino-acid substitution: the seminal PAM matrices of Dayhoff et al. converted curated alignments of close homologues into an evolutionary time-scale [5], while the BLOSUM series by Henikoff & Henikoff mined ungapped blocks of conserved proteins to improve sensitivity for more diverged sequences [7]. These approaches and later profile-based HMM models [6] were derived from pairwise or multiple alignments and remain the gold standard when accurate alignments are available.

Over the last decade, however, high-throughput sequencing has shifted the scale of comparative genomics from dozens to millions of genomes, rendering high computational complexity pipelines (e.g. quadratic-time) increasingly impractical. Consequently, alignment-free techniques that summarize sequences by inexpensive statistics have become indispensable [17, 22]. These approaches most commonly utilize k-mer sets and sketches thereof. Popular tools such as Mash [11], Skmer [13] and more recent sketch-corrected frameworks like Sylph [16] and FracMinHash-based methods [8, 15] can build whole-genome phylogenies, screen metagenomic samples, and compute millions of pairwise point-mutation rate estimates in minutes rather than days.

Despite their empirical success, theoretical understanding of alignment-free estimators has lagged behind practice. Nearly all existing models treat evolution as a pure-substitution process, ignoring insertions and deletions (indels), or else their performance in the presence of indels is often not thoroughly evaluated [12]. When indels are frequent, substitution-only estimators systematically inflate divergence and can misplace taxa—even in otherwise well-resolved trees of primates constructed from k-mer Jaccard similarities [11]. Recent work has quantified how k-mer based statistics are affected by substitutions and are also used to estimate substitution-only mutation rates [8], yet a principled treatment that jointly infers substitution and indel parameters from k-mer statistics is still absent. This omission is particularly significant because indels represent a substantial fraction of genomic variation and play crucial roles in evolution [19]. Such indels cause substitution-only k-mer-based methods to underperform, as disruption of k-mer content by indel events affects at least kk-mers, often leading to overestimates of mutation rates [3, 8].

In this paper we introduce the first closed-form, alignment-free estimators for the three fundamental mutation parameters: substitution rate $p_{s}$, deletion rate $p_{d}$, and mean insertion length d under a model that explicitly incorporates single-nucleotide substitutions, deletions, and geometrically-distributed insertions. This mutation model is closely related to the “indel channel” model of [14]. Starting from elementary counts of unmutated and single-deletion k-mers, we derive algebraic expressions for $p_{s}$, $p_{d}$, and d and prove sub-exponential concentration bounds that guarantee a strong form of consistency as the sequence length grows as detailed in our main contribution: Theorem 10. Simulations on synthetic and real bacterial genomes demonstrates that modeling indels yields markedly more accurate distance estimates than substitution-only approaches. The remainder of this paper is organized as follows: Section 2 defines our mutation model, Section 3 derives basic statistical properties, Section 4 presents our estimators, Section 5 provides theoretical guarantees about these estimators, Section 6 details our implementation, Section 7 presents experimental results, and Section 8 concludes with a discussion of implications and future directions.

## 2 The mutation model

We first describe the mutation model under consideration. Let S be a string over the alphabet {A, C, G, T}, and let L be the number of characters in S. Let $S_{i}$ denote the i-th character in S where 1≤i≤L. We define three operations on a string S as follows:

- Substitute (i): select a character $c\in \{A,C,G,T\}\setminus \{S_{i}\}$ uniformly at random and replace $S_{i}$ by c.
- Delete(i): remove the character $S_{i}$.
- Stay(i): do nothing.
- Insert(i,s): insert string s between $S_{i}$ and $S_{i+1}$ if i<L. If i=L, append s at the end of S instead.

The mutation process takes as input a string S and three parameters $p_{s}$, $p_{d}$, and d, where $0\leq p_{s}$, $p_{d}<1$, $p_{s}+p_{d}<1$, and d≥0. For every position in S, it does the following:

1. For each i, let $a_{i}$ be the operation to be performed at position i. Then, $a_{i}=\text{Substitute}(i)$ with probability $p_{s}$, $a_{i}=\text{Delete}(i)$ with probability $p_{d}$, or $a_{i}=\text{Stay}(i)$ with the remaining probability $1-p_{s}-p_{d}$. (These operations are not performed at this point, but only recorded.)
2. For each i, let $l_{i}\geq 0$ be a sample from a geometric distribution with mean d. Then generate a random string $Q^{\left(i\right)}$ of length exactly $l_{i}$ by drawing each character in $Q^{\left(i\right)}$ from {A, C, G, T} independently and uniformly at random. This is equivalent to sampling a uniformly random string among all strings of length $l_{i}$ with characters in {A, C, G, T}. If $l_{i}=0$, $Q^{\left(i\right)}$ is the empty string.
3. Go through each position in S. For position i, execute the corresponding operation $a_{i}$, and then execute the operation $\text{Insert}(i,Q^{\left(i\right)})$.
4. Return the resulting $S^{\prime }$.

## 3 Preliminary statistics of the mutation process

In this section, we define several quantities whose statistics we use to design an estimator of the parameters of the mutation process. For the remainder of the manuscript, we assume that the mutation process described in Section 2 is applied to string S of length L, with the unknown parameters $p_{s}$, $p_{d}$, and d, and a mutated string $S^{\prime }$ is returned.

**▶ Lemma 1.** Let $L^{\prime }$ be the length of $S^{\prime }$. Then

(1) $$\;E[L^{\prime }]=L\;(1+d-p_{d}),\;\mathrm{Var}[L^{\prime }]=L\left(d(d+1)+p_{d}(1-p_{d})\right).$$

**▶ Lemma 2.** Let $f_{A}$ and ${f_{A}}^{\prime }$ be the number of ‘A’s in S and $S^{\prime }$, respectively. Then,

(2) $$E[{f_{A}}^{\prime }]=f_{A}(1-p_{s}-p_{d})+\frac{p_{s}(L-f_{A})}{3}+\frac{dL}{4}.$$

We can analogously obtain expressions for the expectations of the corresponding counts of ‘C’s, ‘G’s, and ‘T’s in $S^{\prime }$, denoted by ${f_{C}}^{\prime }$, ${f_{G}}^{\prime }$, and ${f_{T}}^{\prime }$ respectively.

Next, we say that a k-mer has no mutations if (a) for all k characters, the mutation process picks a Stay(·) action, and if (b) for all k−1 intermediate positions of the k-mer, the mutation process does not insert anything. Let 𝒩 be the number of such k-mers. Let 𝒦 be the number of all k-mers in S.

**▶ Lemma 3.** $E[𝒩]=𝒦\;{\left(1-p_{s}-p_{d}\right)}^{k}\frac{1}{{\left(d+1\right)}^{k-1}}$.

The last quantities we will use correspond to the number of k-mers with a single kind of mutation. Let 𝒮 be the number of k-mers with a single substitution, and no other mutations, let 𝒟 be the number of k-mers with a single deletion and no other mutations, and let ℐ be the number of k-mers with a single insertion and no other mutations.

**▶ Lemma 4.** The expectations of 𝒮, 𝒟, and ℐ are given by:

(3) $$E[𝒮]=𝒦\;k\;{\left(1-p_{s}-p_{d}\right)}^{k-1}\;p_{s}\;\frac{1}{{\left(d+1\right)}^{k-1}}.$$

(4) $$E[𝒟]=𝒦\;k\;{\left(1-p_{s}-p_{d}\right)}^{k-1}\;p_{d}\;\frac{1}{{\left(d+1\right)}^{k-1}}.$$

(5) $$E[ℐ]=𝒦\;(k-1)\;{\left(1-p_{s}-p_{d}\right)}^{k}\;\frac{d}{{\left(d+1\right)}^{k}}.$$

## 4 Estimating $p_{s}$, $p_{d}$ and d

We describe next our estimators for the parameters of the mutation model $p_{s}$, $p_{d}$, and d. We derive estimators for these rates based on the statistics defined in Section 3. Recall that S is the known input string to the mutation process, and $S^{\prime }$ is the resulting random string or observation.

Our mutation model assumes $p_{s}+p_{d}<1$, and d≥0. In addition, we assume that L≥k which implies that E[𝒩]>0. In reality, strings are typically much longer than k-mer sizes and therefore this is a reasonable assumption. We will also assume that $p_{d}>0$, which implies that E[𝒟]>0. We will handle the $p_{d}=0$ case separately.

From Lemmas 3 and 4 we get

(6) $$\frac{E[𝒟]}{E[𝒩]}=\frac{k\;p_{d}}{1-p_{s}-p_{d}}.$$

Reorganizing (1), (2) and (6), we have the following system of linear equations with variables $p_{s}$, $p_{d}$, and d.


(7) $$-p_{d}+d=\frac{E[L^{\prime }]}{L}-1$$

(8) $$\frac{L-4f_{A}}{3}\;p_{s}-f_{A}\;p_{d}+\frac{L}{4}d=E[{f_{A}}^{\prime }]-f_{A}$$

(9) $$p_{s}+\left(\frac{kE[𝒩]}{E[𝒟]}+1\right)p_{d}=1.$$

Solving this system of equations, we obtain that:

$$p_{s}=3\frac{k\;E[𝒩]\left(E[L^{\prime }]-L+4f_{A}-4E[{f_{A}}^{\prime }]\right)+E[𝒟]\left(E[L^{\prime }]-4E[{f_{A}}^{\prime }]\right)}{\left(4f_{A}-L\right)\left(E[𝒟]+4kE[𝒩]\right)}.$$

Given S and $S^{\prime }$, we can observe $L^{\prime }$, ${f_{A}}^{\prime }$, 𝒩, and 𝒟. By replacing the expectations above with these observations, we obtain our estimators for the parameters of mutation model. That is,

(10) $${\hat{p}}_{s}=3\frac{k\;𝒩\left(L^{\prime }-L+4f_{A}-4f_{A}\prime \right)+𝒟\left(L^{\prime }-4{f_{A}}^{\prime }\right)}{\left(4f_{A}-L\right)\left(𝒟+4k𝒩\right)},$$

(11) $${\hat{p}}_{d}=\frac{𝒟\;[3(L^{\prime }-4f_{A}\prime )+(L-4f_{A})]}{\left(L-4f_{A})(𝒟+4k𝒩\right)},\text{and}$$

(12) $$\hat{d}=\frac{L^{\prime }}{L}-1+\frac{𝒟\;[3(L^{\prime }-4{f_{A}}^{\prime })+(L-4f_{A})]}{\left(L-4f_{A})(𝒟+4k𝒩\right)}.$$

We briefly comment on our choice of estimators for the mutation model parameters, as various statistical approaches based on a different set of observables could yield a different set of estimators. For example, we considered a variant of the estimators based on the counts of k-mers with a single insertion, deletion, or substitution (i.e., ℐ, 𝒮, and 𝒟). These quantities contain enough information to estimate the mutation parameters (specifically, by solving the non-linear system of equations given by Lemma 4), and the resulting estimators performed quite well in real data. However, establishing theoretical guarantees for these estimators proved challenging, as they were defined as roots of degree-k polynomials . Our current estimators address this theoretical limitation as they involve solely linear equations. As we shall see in Section 7, the performance of our estimators in real data is strong, and they strike a more favorable balance by offering both reasonable accuracy and rigorous theoretical analysis.

▶ Remark 5. We briefly describe estimators for the case when $p_{d}=0$, which corresponds to a strictly simpler mutation process without deletions. In this case, we would observe 𝒟=0. When this happens, (9) becomes undefined, and therefore, we can only use (7) and (8). Plugging in $p_{d}=0$ into (7) and (8), we obtain the estimators:

(13) $${\hat{p}}_{s}=\frac{3({f_{A}}^{\prime }-f_{A}+L∕4-L^{\prime }∕4)}{L-4f_{A}},$$

(14) $${\hat{p}}_{d}=0,\text{and}$$

(15) $$\hat{d}=\frac{L^{\prime }}{L}-1.$$

We note that we did not encounter this case in our simulation experiments. Nevertheless, we include this case for completeness.

## 5 Theoretical results for ${\hat{p}}_{s}$, ${\hat{p}}_{d}$, and $\hat{d}$

In this section, we provide theoretical guarantees for the estimators from Section 4; i.e., (10), (11), and (12). In particular, we show that our estimates of $p_{s}$, $p_{d}$, and d are not only consistent estimators, but also that they are tightly concentrated in a symmetric interval around the true values of $p_{s}$, $p_{d}$, and d. We provide the bias analysis for ${\hat{p}}_{s}$ by proving asymptotically tight concentration bounds for 𝒩, 𝒟, and $L^{\prime }-4{f_{A}}^{\prime }$. We then describe how to obtain analogous results for ${\hat{p}}_{d}$ and $\hat{d}$.

The concentration bounds for 𝒩 and 𝒟 stem from the fact that, from the perspective of the mutation process, k-mers located more than k positions apart are independent of each other.

**▶ Lemma 6.** For any δ∈(0,1):

$$\text{Pr}\left[|𝒩-E[𝒩]|\geq \delta E[𝒩]\right]\leq 2k\;\text{exp}\left\{-\frac{\delta^{2}E[𝒩]}{3k}\right\}.$$

**▶ Lemma 7.** For any δ∈(0,1):

$$\text{Pr}\left[|𝒟-E[𝒟]|\geq \delta E[𝒟]\right]\leq 2k\;\text{exp}\left\{-\frac{\delta^{2}E[𝒟]}{3k}\right\}.$$

We prove a similar result for $P=L^{\prime }-4{f_{A}}^{\prime }$ by noting that P can expressed as a sum of independent sub-exponential random variables. Our concentration bound for P is as follows.

**▶ Lemma 8.** Let $K_{1}=1∕\text{min}\{\frac{1}{d+1},\frac{1}{8}\}$. Then, there exist absolute constants $c_{1}$, $c_{2}>0$ such that the following holds for any δ>0:

$$\text{Pr}\left[|P-E[P]|\geq 3\delta \right]\leq 2\;\text{exp}\left\{-\frac{\delta^{2}}{25f_{A}}\right\}+2\;\text{exp}\left\{-\frac{\delta^{2}}{3(L-f_{A})}\right\}+2\;\text{exp}\left\{-c_{1}\;\text{min}\left(\frac{\delta^{2}}{{c_{2}}^{2}{K_{1}}^{2}},\frac{\delta }{c_{2}K_{1}}\right)\right\}.$$

Finally, we prove that $L^{\prime }$ is also strongly concentrated around $E[L^{\prime }]$.

**▶ Lemma 9.** For any δ∈(0,1):

(16) $$\text{Pr}\left[|L^{\prime }-E[L^{\prime }]|\leq \delta (Ld+Lp_{d})\right]\geq 2\;\text{exp}\left\{-\frac{L\delta^{2}d^{2}}{2(d+1-\delta d)(d+1)}\right\}+2\;\text{exp}\left\{-\frac{Lp_{d}\delta^{2}}{3}\right\}.$$

We can piece the results of these lemmas together and prove the following result.

**▶ Theorem 10.** Suppose $4f_{A}\leq L$ and $\frac{4}{3}p_{s}+p_{d}\leq 1$. Then, for sufficiently small δ>0, there exists constants $c_{1}$, $c_{2}>0$ such that

$$\text{Pr}[|{\hat{p}}_{s}-p_{s}|\geq 4\delta ]\leq 6k\;\text{exp}\left\{-\frac{\delta^{2}E[𝒩]}{3k}\right\}4k\;\text{exp}\left\{-\frac{\delta^{2}E[𝒟]}{3k}\right\}+\;\;+2\;\text{exp}\left\{-\frac{\delta^{2}\mu_{P}^{2}}{225f_{A}}\right\}+2\;\text{exp}\left\{-\frac{\delta^{2}\mu_{P}^{2}}{27(L-f_{A})}\right\}+2\;\text{exp}\left\{-c_{1}\;\text{min}\left(\frac{\delta^{2}\mu_{P}^{2}}{{c_{2}}^{2}},\frac{\delta \mu_{P}}{c_{2}}\right)\right\}.$$

The requirement that $4f_{A}\leq L$ in this theorem does not restrict generality: aside from equal nucleotide frequency (where the estimators are naturally invalid), at least one character c∈{A,C,T,G} must satisfy $4f_{c}\leq L$. In addition, the assumption that $\frac{4}{3}p_{s}+p_{d}\leq 1$ holds when $p_{s}$ and $p_{d}$ are small (e.g., $p_{s}\leq 1∕4$ and $p_{d}\leq 1∕4$ suffices) which is the case most frequently encountered in practice.

We can obtain analogous results for ${\hat{p}}_{d}$ and $\hat{d}$ using the same approach. As such, Theorem 10 is our central theoretical result, establishing that the estimators developed in Section 4 are sound under the assumed conditions. Before discussing our implementation and presenting the experimental results, we comment on the error probability in Theorem 10. This probability is small when each of the terms in the sum are small. Since each of these terms decays (at least) exponentially with δ times an expectation, they will all generally be small. In particular:

- The probabilistic guarantees for the estimators are asymptotically formulated. Consequently, as the lengths of the strings S and $S^{\prime }$ increase, the probability of errors decreases, and the estimators become more accurate.
- Assuming $p_{s}+p_{d}<1$, and assuming the strings S and $S^{\prime }$ are sufficiently long, E[𝒩] is always positive, and the first error term in the sum is sufficiently small.
- Assuming $p_{s}+p_{d}<1$, and assuming the strings S and $S^{\prime }$ are sufficiently long, if $p_{d}=0$, then we would observe 𝒟=0. In such a case, the theorem does not apply and we would need to use (13) to (15) to compute ${\hat{p}}_{s}$ and $\hat{d}$, as detailed in Remark 5. We note that in such a case, the fact that ${\hat{p}}_{s}$ is close to $p_{s}$ with high probability follows easily from Lemma 8.

## 6 Implementation details

Our estimators for the three rates require counting the number of k-mers with single deletion, 𝒟, and the number of k-mers with no mutation, 𝒩. Counting these two quantities is not trivial and can be considered an interesting problem in and of itself. We therefore implemented an ad hoc solution to count 𝒟 and 𝒩 given the two strings S and $S^{\prime }$. The steps to count 𝒟 and 𝒩 are as follows.

We start by extracting all k-mers in S, and building a de Bruijn graph using these k-mers. We then extract the unitigs from this graph. Let the set of unitigs computed from S be U. We also compute the unitigs in $S^{\prime }$ in a similar manner and call this $U^{\prime }$. We next take an arbitrary unitig u from U, and align every unitig in $U^{\prime }$ with u. We then use the alignment to look at all windows of length k, and count 𝒟 and 𝒩 accordingly for u. We repeat this for all u∈U, and accumulate the measurements from individual u’s into a single global count.

The motivation behind using unitigs is that if there is an isolated mutation, and if the mutation is in the first or the last position of a k-mer, then there is no way to understand if the mutation is a substitution, an insertion, or a deletion only from the k-mers. The only way to resolve this ambiguity is to scan beyond the context of k characters – and unitigs are a natural way to do this.

In our implementation, we use semi-global alignment to allow part of one unitig to be aligned with the other. We build the de Bruijn graph and the unitigs using cuttlefish [10], and compute alignments using edlib [18]. The core goal of our implementation of these steps was not to make it efficient, but rather to obtain a working solution. As such, we leave an efficient way to compute 𝒟 and 𝒩 an open research question.

## 7 Experiments and results

In this section, we present a series of experiments to evaluate the performance of the estimators detailed in Section 4. As discussed earlier, these estimators are sensitive to several input parameters, including k-mer size, sequence length, and the fraction of ‘A’ characters in the sequence. Sections 7.1 through 7.3 explore the sensitivity of the estimators with respect to these parameters. In Sections 7.4 and 7.5, we estimate mutation rates across a wide range of known rate combinations. And finally, in Section 7.6, we demonstrate that our estimated substitution rate outperforms estimates obtained under a substitution-only mutation model. For the experiments in Sections 7.1 through 7.4, the original sequence is a randomly generated synthetic sequence. In these cases, we compute the number of k-mers containing a single deletion and the number of unmutated k-mers directly from the known mutation process. For the experiments in Sections 7.5 and 7.6, we use real reference genomes as the original sequences. In these cases, the two types of k-mer counts are obtained using the steps described in Section 6.

### 7.1 Sensitivity of the estimators to k-mer lengths

**Figure 1:**
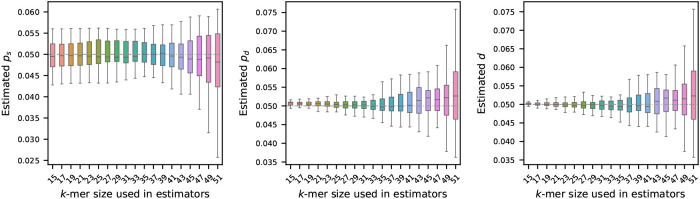
Effect of k-mer length on mutation rate estimation (true rates set to 0.05). A synthetic genome of 1 million nucleotides, mutated genomes were generated by setting $p_{s}$, $p_{d}$, and d=0.05 – shown by the gray dashed horizontal line. Estimated rates were then computed using a range of k-mer sizes. Each boxplot shows the variability in estimation across 20 simulations, with error bars showing one standard deviation. The plots show that the estimators become more accurate and more precise for shorter k-mers.

We begin our analysis by examining how the choice of k-mer length affects the estimation of the mutation rates. To investigate this effect, we first generated a synthetic reference sequence of 1 million nucleotides, randomly sampling bases with fixed frequencies: 30% ‘A’, 20% ‘C’, 20% ‘G’, and 20% ‘T’. From this reference, we simulated 30 mutated sequences, independently from each other, using the mutation model described in Section 2. For each of these mutated sequences, we estimated mutation rates using the estimators defined in Section 4 using a range of values for k. The results of this analysis are summarized in Figure 1.

As illustrated, the choice of k has a substantial impact on the stability of the estimators. In particular, longer k-mers tend to produce estimates with higher variability. This behavior is consistent with the known sensitivity of k-mers to mutations: since a single mutation can disrupt up to k consecutive k-mers, the longer the k-mer, the more susceptible it becomes to such perturbations. Our theoretical result in Theorem 10 also captures this: with a larger k, the error probabilities become larger, and the probabilistic guarantee for the estimators’ performances decreases accordingly.

Interestingly, for the estimator of substitution probability $p_{s}$, we observe that the variability in the estimated values does not change significantly from 15 to 39. The reason behind this behavior is not immediately clear and warrants further investigation. It is possible that incorporating the number of k-mers with a single substitution 𝒮 in the estimators may correct this behavior, but additional analyses are required to substantiate this hypothesis.

### 7.2 Sensitivity of the estimators to sequence length

To investigate how the length of the original sequence 𝒮 influences estimation of the mutation rates, we simulated synthetic genomes ranging from 10K to 1M nucleotides in length. For each genome length, we generated 10 independent synthetic sequences to capture variability due to random sampling. The nucleotide composition of each sequences was fixed, with the frequencies of ‘A’, ‘C’, ‘G’, and ‘T’ set to 0.3, 0.2, 0.2, and 0.2, respectively. For each synthetic sequence, we generated its mutated version by running the mutation process described in Section 2, setting each of $p_{s}$, $p_{d}$, and d to 0.05. We then estimated the mutation rates using the estimators outlined in Section 4 for three k-mer sizes: k=21,31,41.

**Figure 2:**
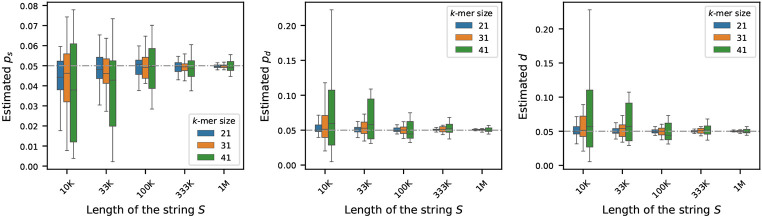
Effect of sequence length on mutation rate estimation (true rates set to 0.05). For synthetic genomes of varying lengths, mutated genomes were generated by setting $p_{s}$, $p_{d}$, and d=0.05 – shown by the gray dashed horizontal line. Estimated rates were computed using three k-mer sizes: 21, 31, and 41. Each boxplot shows the variability in estimation across 20 simulations. The plots show that the estimators become more accurate and more precise for longer sequences.

Figure 2 displays the estimated rates across the varying sequence lengths. As shown, the estimators are less stable for shorter sequences. However, with longer sequences, the estimators yield more accurate results – a trend expected from our core theoretical result in Theorem 10, which states that the associated error is asymptotically vanishing in L, the length of the string S: as L increases, the number of k-mers 𝒦 increases, and therefore the probability of error decreases, leading to a more precise estimation.

### 7.3 Sensitivity of the estimators to base composition

**Figure 3:**
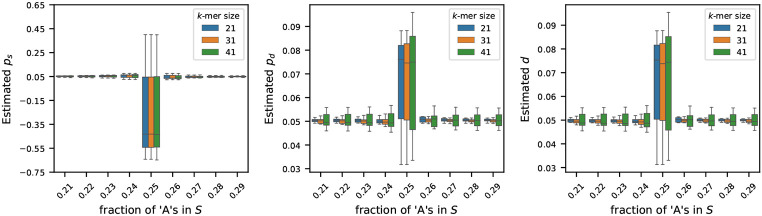
Effect of nucleotide composition on mutation rate estimation (true rates set to 0.05). For synthetic genomes of length L=1M, the fraction of ‘A’s is varied from 0.21 to 0.29, and the frequencies of ‘G’s, ‘C’s, and ‘T’s are set equally. For each setting, the mutated string $S^{\prime }$ was generated by setting $p_{s}$, $p_{d}$, d=0.05. Estimated rates were computed for three k-mer sizes: 21, 31, and 41. Each boxplot shows the variability in estimation across 20 simulations. The results show that the estimators generally work well for all three k-mer sizes, except when $f_{A}\approx L∕4$, in which case the estimators become unstable – as predicted by Theorem 10.

We generated synthetic genomes of 1M nucleotides to investigate how the fraction of ‘A’ characters affects the estimation of the mutation rates. We varied the fraction of ‘A’s from 21% to 29% in increments of 1%. For each fraction of ‘A’s, we set the frequency of ‘C’s, ‘G’s, and ‘T’s equally. For each preset fraction of ‘A’s, we generated 10 random genomes to capture stochastic variation. For each of these genomes, we generated its mutated version using the mutation process described in Section 2, setting each of $p_{s}$, $p_{d}$, and d to 0.05. We then estimated the mutation rates using the estimators described in Section 4 using three k-mer sizes: k=21,31 and 41. Figure 3 shows the sensitivity of the estimators to the fraction of ‘A’s in the original string S.

We observe that the estimators ${\hat{p}}_{s}$, ${\hat{p}}_{d}$, and $\hat{d}$ work reasonably well to estimate the true rates when the frequency of ‘A’ characters, $f_{A}$ is not L∕4. On the contrary, when the fraction of ‘A’s is exactly 25% in the original string S, the estimator gives inaccurate values, some of which are even negative (see estimated values of $p_{s}$). This behavior is captured in Theorem 10: when $L-4f_{A}=0$, $\mu_{P}=E[P]=0$, and the probabilistic guarantees become unbounded. We only get meaningful guarantees of consistency when $4f_{A}$ is strictly smaller than L. While Theorem 10 does not guarantee consistency when $4f_{A}\;>\;L$, this does not restrict generality, as explained in Section 4, and as demonstrated by the estimators’ performances when the fraction of ‘A’s is larger than 1/4.

### 7.4 Estimating rates from a randomly generated synthetic sequence

After testing our estimators for varying k-mer lengths, sequence lengths, and base compositions, we next turn to estimating mutation rates by varying the true rates across a range of values. To do this, we generated a synthetic reference genome of 1 million base pairs, fixing the base composition at 30% ‘A’, and 20% each for ‘C’, ‘G’, and ‘T’. Using the mutation model described in Section 2, we then simulated mutated genomes from the synthetic reference by varying the mutation rates $p_{s}$, $p_{d}$, and d across the values {0.01, 0.02, 0.03, 0.04, 0.05}. For every parameter combination, we generated 10 independent mutated genomes to capture stochastic variability. We then estimated the mutation rates using the estimators detailed in Section 4 for each of these mutated genomes.

**Figure 4:**
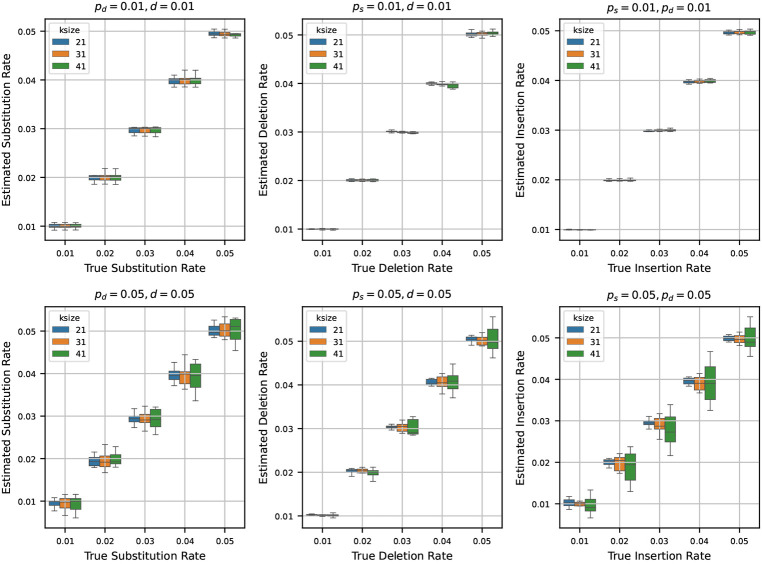
Estimated mutation rates versus true values, where the original string is a synthetic sequence. Each subplot corresponds to a case where two mutation rates are fixed (either at 0.01 or 0.05) and the third is varied from 0.01 to 0.05. Each boxplot shows the variability in estimation across 10 simulations. The results show that the rate estimation is very accurate when the other two rates are small, and is reasonably accurate when the other two rates are larger.

In Figure 4, we show two sets of results:

- **Fixed low rates (0.01):** Fixing two of the rates at 0.01, we show the estimates of the third rate as the true rate varies from 0.01 to 0.05. We repeat this process independently for $p_{s}$, $p_{d}$, and d.
- **Fixed high rates (0.05):** Fixing two of the rates at a higher value of 0.05, we also show the estimates of the third rate as the true rate varies from 0.01 to 0.05. Again, we repeat this for all three rates.

When we set the other two rates to 0.01 and estimate the third rate, we observe that the estimated rates are highly accurate across all trials. In many cases, the boxplots of the estimates nearly vanish due to minimal variance, indicating tight clustering around the true values. This trend remains consistent across multiple k-mer sizes, suggesting that the estimators are robust at low rates of mutation.

In contrast, when we fix the other two rates at 0.05 and estimate the third rate, the accuracy of the estimation decreases slightly. While the estimates still track the true values reasonably well, the variance increases, and the boxplots become more prominent. Notably, the median estimate remains close to the true rate in most settings, which indicates that the estimators retain their central tendency even under higher mutation rates. However, for larger k-mer sizes, we observe increased variability in the estimates – an effect that mirrors our earlier observations in Section 7.1, where longer k-mers resulted in decreased precision of the estimators.

### 7.5 Estimating rates from real sequences

Having the estimators tested for a synthetic reference, we next estimate rates from a real genome sequence. For this set of experiments, we used the reference assembly of Staphylococcus aureus (subspecies: aureus USA300_TCH1516), which has 2.8 million nucleotides. We simulated mutated sequences from this reference by running the mutation process described in Section 2 by varying the mutation rates $p_{s}$, $p_{d}$, and d from 0.01 to 0.05. Similar to Section 7.4, we generated 10 independent mutated sequences for each combination of $p_{s}$, $p_{d}$, and d to capture stochastic variability. We then estimated the mutation rates using the estimators outlined in Section 4 for each of these simulated sequences.

**Figure 5:**
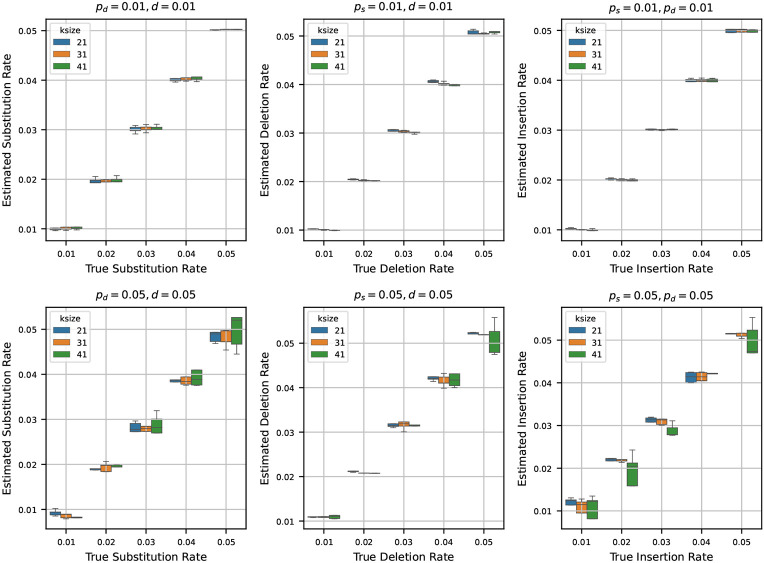
Estimated mutation rates versus true values, where the original string is the reference genome of Staphylococcus (length 2.8 million). Each subplot corresponds to a case where two mutation rates are fixed (either at 0.01 or 0.05) and the third varies from 0.01 to 0.05. Each boxplot shows the variability in estimation across 10 simulations. Estimated mutation rates closely match true values when other rates are low, but estimation becomes less precise under high total mutation rates (>10%) due to increased difficulty in real genomic contexts.

Figure 5 shows the estimated mutation rates plotted against the true rates that were used to run the mutation process. We observe that the results shown in Figure 5 are consistent with previously discussed results. Specifically, when estimating a given mutation rate while keeping the other two rates low (0.01), the corresponding estimator performs with high precision, closely tracking the true value. On the other hand, when the estimation is carried out with the other two mutation rates set to higher values (0.05), the estimates appear more confounded. This is likely due to the increased difficulty of accurately counting k-mers with a single deletion ((𝒟)) or no mutation ((𝒩)) in a real genomic context. Notably, these experiments involve challenging conditions, with total mutation rates exceeding 10%. Despite this, the estimators yield reasonably accurate results, indicating potential for practical effectiveness.

### 7.6 Comparison with substitution rates estimated using simple mutation model

We conclude the experiments section by contrasting the substitution rates estimated using Equation (10) with substitution rates estimated considering a simple mutation model. We use the statistics of k-mers developed in a recent work [1] to estimate substitution rates under a simple mutation model. The simple mutation model captures only substitutions, and no insertions or deletions. Consequently, we can only compute substitution rates considering this simple model. Henceforth, we refer to the simple mutation model as SMM.

**Figure 6:**
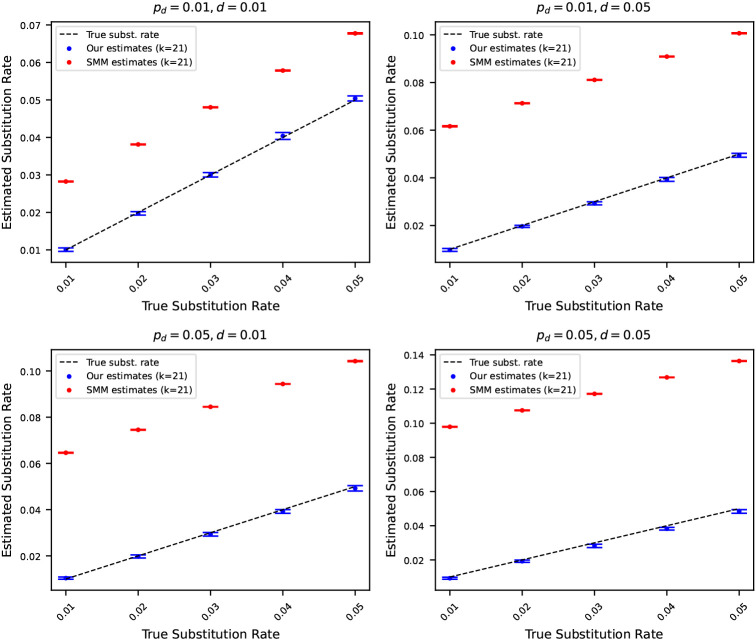
Estimated substitution rates versus true substitution rates, where the original string is the reference genome of Staphylococcus (length 2.8 million). Rates were estimated using Equation (10) and using a simple mutation model (SMM) that only considers substitution. Each subplot corresponds to a case where $p_{d}$ and d are fixed at either 0.01 or 0.05. The points show the average of 10 estimates, and the error bars show one standard deviation. The dashed black line corresponds to the true substitution rates. Estimated substitution rates using our method closely match true rates, whereas SMM overestimates due attributing to substitutions mismatching k-mers originating from insertions and deletions.

We estimated the substitution rates using the SMM for the same simulated mutated sequences described in Section 7.5. In Figure 6, we show the estimated substitution rates using our estimators (Equation (10)) and using SMM. The results highlight that the substitution rates estimated using the estimator we developed track the true substitution rates accurately. On the other hand, the substitution rates estimated using SMM make a gross overestimation. This is because the SMM does not consider indels, and therefore, the effects of all three mutation rates are subsumed in the single substitution rate we get using the SMM. As such, a simple mutation model cannot disentangle the distinct contributions of substitution, insertion, and deletion rates. In contrast, the mutation model we introduce effectively decomposes these components, enabling more accurate and meaningful estimation of individual mutation rates.

## 8 Conclusions

We have presented a mutation model that accommodates single-nucleotide substitutions, as well as insertions and deletions while retaining enough mathematical structure to admit closed-form rate estimators derived solely from k-mer statistics. From this model, we obtained unbiased, purely algebraic estimators for the three elementary mutation rates: $p_{s}$, $p_{d}$, and d; proved relatively tight sub-exponential concentration bounds that cumulatively guarantee consistency as sequence length grows. We also identified regimes in which the estimation becomes ill-conditioned (i.e. large k, $p_{d}=0$, or sequence composition with 25% ‘A’). These results establish a bridge between sequence evolution and combinatorial word statistics, thus providing additional tools for theoretical algorithmic computational biology.

In our prototype implementation, we demonstrated that our estimates remain accurate on simulated evolution of real genomes, and outperforms a substitution-only simple mutation model by avoiding spurious attribution of indel signals. While naive counting of unmutated and single-deletion k-mers sufficed to show practical accuracy of our estimators, this raises and interesting open problem: estimating the number of these unmutated and single-deletion k-mers in efficiently for large scale data sets.

Several directions invite further investigation. First, incorporating the count 𝒮 of single-substitution k-mers may illuminate why $p_{s}$ remains relatively stable even for moderately large k. Second, our framework can extend to heterogeneous or context-dependent rates by replacing global expectations with position-specific covariates. Third, coupling our estimators with sketch-based distance measures (such as in [8]) may provide a theory-backed avenue for larger scale applications such as phylogenetic placement in the presence of high indel activity. Finally, a more thorough investigation on real genomic data (where the unitig-based approach we used in the practical implementation starts to become infeasible) will be necessary to understand the utility of the mutation estimates in practice.

In summary, by utilizing probabilistic modeling and concentration inequalities, we provide a theoretical foundation and initial practical implementation for quantifying a relatively complex mutation process parameters directly from k-mers. We anticipate that these ideas will continue to inform new alignment-free computational biology tasks, particularly relevant as sequencing data continues to outpace traditional alignment-based paradigms.

## Supplementary Material

1
