# Estimation of substitution and indel rates via *k* -mer statistics

**DOI:** 10.1101/2025.05.14.653858

**Published:** 2025-06-21

**Authors:** Mahmudur Rahman Hera, Paul Medvedev, David Koslicki, Antonio Blanca

## Abstract

Methods utilizing *k* -mers are widely used in bioinformatics, yet our understanding of their statistical properties under realistic mutation models remains incomplete. Previously, substitution-only mutation models have been considered to derive precise expectations and variances for mutated *k* -mers and intervals of mutated and non-mutated sequences. In this work, we consider a mutation model that incorporates insertions and deletions in addition to single-nucleotide substitutions. Within this framework, we derive closed-form *k* -mer-based estimators for the three fundamental mutation parameters: substitution, deletion rate, and insertion rates. We provide theoretical guarantees in the form of concentration inequalities, ensuring accuracy of our estimators under reasonable model assumptions. Empirical evaluations on simulated evolution of genomic sequences confirm our theoretical findings, demonstrating that accounting for insertions and deletions signals allows for accurate estimation of mutation rates and improves upon the results obtained by considering a substitution-only model. An implementation of estimating the mutation parameters from a pair of fasta files is available here: github.com/KoslickiLab/estimate_rates_using_mutation_model.git . The results presented in this manuscript can be reproduced using the code available here: github.com/KoslickiLab/est_rates_experiments.git .

**Supplementary Material:**

The full version of this manuscript, including all proofs, is available on the BioRxiv: https://doi.org/10.1101/2025.05.14.653858

**Funding:**

This material is based upon work supported by the National Science Foundation under Grant No. DBI2138585. Research reported in this publication was supported by the National Institute Of General Medical Sciences of the National Institutes of Health under Award Number R01GM146462. The content is solely the responsibility of the authors and does not necessarily represent the official views of the National Institutes of Health.

## Full Text Availability

The license terms selected by the author(s) for this preprint version do not permit archiving in PMC. The full text is available from the preprint server.

