# Supplementary material for: Estimation of substitution and indel rates via k-mer statistics
Source: bioRxiv. 2025 Jul 27:2025.05.14.653858. Preprint. [Version 4] doi: 10.1101/2025.05.14.653858 (PMC12132262; doi:10.1101/2025.05.14.653858)
Supplement: 1 [file NIHPP2025.05.14.653858V4-supplement-1.pdf]

bioRxiv preprint doi: https://doi.org/10.1101/2025.05.14.653858; this version posted July 27, 2025. The copyright holder for this preprint (which was not certified by peer review) is the author/funder, who has granted bioRxiv a license to display the preprint in perpetuity. It is made available under aCC-BY 4.0 International license.



## A Standard concentration inequalities

This section gathers several definitions and standard concentration inequalities that we use in our proofs. Certain sums of independent random variables exhibit sharp concentration, and this is captured by the following standard inequalities. The first one is the Chernoff bound for sums of independent and identically distributed Bernoulli random variables [1, 4].

▶ **Lemma 8.** *Let $X_1, \ldots, X_n$ be a sequence of independently and identically distributed Bernoulli random variables. Consider $X = \sum_{i=1}^n X_i$ and let $\mathbf{E}[X] = \mu$. Then, the following three inequalities hold:*

$$\Pr[X \geq (1+\delta)\mu] \leq e^{-\delta^2\mu/(2+\delta)}, \qquad 0 \leq \delta. \tag{21}$$

$$\Pr[X \leq (1-\delta)\mu] \leq e^{-\delta^2\mu/2}, \qquad 0 < \delta < 1. \tag{22}$$

$$\Pr[|X-\mu| \geq \delta\mu] \leq 2e^{-\delta^2\mu/3}, \qquad 0 < \delta < 1. \tag{23}$$

A more general inequality that applies to sums of independent and bounded random variables is Hoeffding's inequality [12].

▶ **Lemma 9.** *Let $X_1, \ldots, X_n$ be a sequence of independent random variables taking values in $[a, b]$. Let $X = \sum_{i=1}^n X_i$, and $\mathbf{E}[X] = \mu$. Then, for any $\delta > 0$:*

$$\Pr\left[|X-\mu| \geq \delta\right] \leq 2\exp\left(-\frac{2\delta^2}{n(b-a)^2}\right).$$

We will also analyze sums of certain *unbounded* random variables. For this, we recall the definition of sub-exponential random variables. For a comprehensive survey about this type of random variables, the reader is referred to [24, Chapter 2.7].

▶ **Definition 10.** *Let $a, b$ be fixed positive real numbers. A random variable $X$ such that $\mathbf{E}[X] = 0$ is called $(a, b)$-sub-exponential if for all $t \geq 0$*

$$\Pr\Big[|X| \geq t\Big] \leq ae^{-t/b}.$$

For sums of $(a, b)$-sub-exponential random variables, the following version of Bernstein's inequality can be derived following the argument in the proof of [24, Theorem 2.8.1].

▶ **Lemma 11.** *Let $a, b$ be fixed positive real numbers and let $X_1, \ldots, X_n$ be a sequence of independent $(a, b)$-sub-exponential random variables. Let $X = \sum_{i=1}^n X_i$. Then, for every $t \geq 0$:*

$$\Pr\left[|X| \geq t\right] \leq 2\exp\left(-\min\left(\frac{t^2}{8ne^{2(ab+1)}}, \frac{t}{2e^{ab+1}}\right)\right).$$

For completeness, we provide a proof of this concentration inequality in Appendix B. We derive and use this variant of Bernstein's inequality instead of the one in [24, Theorem 2.8.1] since that one does not track the dependency on $a$ and $b$ in the tail bound.

We will also use the following lemma to combine concentration inequalities, with a proof given in Appendix C.

▶ **Lemma 12.** *For all random variables $X_1, \ldots, X_n$ and numbers $t_1, \ldots, t_n$,*

$$\Pr\left[\left|\sum_{i=1}^n X_i\right| \geq \sum_{i=1}^n t_i\right] \leq \sum_{i=1}^n \Pr\left[|X_i| \geq t_i\right].$$

bioRxiv preprint doi: https://doi.org/10.1101/2025.05.14.653858; this version posted July 27, 2025. The copyright holder for this preprint (which was not certified by peer review) is the author/funder, who has granted bioRxiv a license to display the preprint in perpetuity. It is made available under aCC-BY 4.0 International license.



## B Proof of main result

▶ **Lemma 1.**

$$\mathbf{E}[L'] = L\,(1 + g - p_d), \tag{1}$$

$$\mathbf{E}[N] = L_0\,(1 - p_s - p_d)^{\,k}\,\frac{1}{(g+1)^{\,k-1}}. \tag{2}$$

$$\mathbf{E}[D] = L_0\,k\,(1 - p_s - p_d)^{k-1}\,p_d\,\frac{1}{(g+1)^{\,k-1}}. \tag{3}$$

$$\mathbf{E}[c_A{}'] = c_A(1 - p_s - p_d) + \frac{p_s(L - c_A)}{3} + \frac{gL}{4}. \tag{4}$$

$$\mathbf{E}[P] = (L - 4c_A)(1 - \frac{4}{3}p_s - p_d) \tag{5}$$

**Proof.** *Proof of* (1)*:* Let $W_i$ be an indicator random variable that is 1 in the event that character $S_i$ was deleted. Then $\mathbf{E}[L'] = \mathbf{E}[L + \sum_{i=1}^{L}(W_i + I_i)] = L + \sum_{i=1}^{L}(\mathbf{E}[W_i] + \mathbf{E}[I_i]) = L + L(p_d + g)$.

*Proof of* (2)*:* Let $N_i$ be the indicator that $k$-span $K_i$ has no mutations. This happens when each of its $k$ positions has a Stay operation and there are no string inserted inside the $k$-span, i.e. $l_j = 0$ for each $j \in (i+1, \ldots, i+k-1)$. This happens with probability $(1 - p_s - p_d)^k/(g+1)^{k-1}$. The result follows since $N = \sum_{i=1}^{L_0} N_i$.

*Proof of* (3)*:* Let $D_i$ be an indicator that $K_i$ has a single deletion and no other mutation. This happens when there are no strings inserted inside the $k$-span, one position has a deletion, and $k-1$ other positions have a Stay operation. The single deletion operation could be at any of the $k$ positions. Then, $\mathbf{E}[D_i] = k(1 - p_s - p_d)^{k-1} p_d/(g+1)^{k-1}$. The result follows since $D = \sum_{i=1}^{L_0} D_i$.

*Proof of* (4)*:* Let $X_i$ be an indicator $S_i$ is not deleted or substituted, which happens with probability $1 - p_s - p_d$. Let $Y_i$ bet the indicator $S_i$ was substituted into ‘A’, which happens with probability $p_s/3$. Let $Z_i$ be the number of ‘A’s in the inserted string associated with position $i$. We have that

$$\mathbf{E}[Z_i] = \sum_{\ell \geq 0} \mathbf{E}[Z_i \mid I_i = \ell]\,\Pr[I_i = \ell] = \sum_{\ell \geq 0} \frac{\ell}{4}\,\Pr[I_i = \ell] = \frac{g}{4}. \tag{24}$$

We can express $c_A{}'$ as: $c_A{}' = \sum_{i:S_i=\text{‘A’}} X_i + \sum_{i:S_i\neq\text{‘A’}} Y_i + \sum_{i=1}^{L} Z_i$, and thus get the claimed result that

$$\mathbf{E}[c_A{}'] = \sum_{i:S_i=\text{‘A’}} \mathbf{E}[X_i] + \sum_{i:S_i\neq\text{‘A’}} \mathbf{E}[Y_i] + \sum_{i=1}^{L} \mathbf{E}[Z_i] = c_A(1 - p_s - p_d) + (L - c_A)p_s/3 + gL/4.$$

*Proof of* (5)*:* It follows directly by linearity of expectation and (1) and (4). ◀

▶ **Lemma 4.** *Suppose that* $4c_A < L$*. For any* $\delta \in (0,1)$*, all the following hold:*

$$\Pr\left[\left|N - \mathbf{E}[N]\right| \geq \delta\mathbf{E}[N]\right] \;\leq\; 3k\exp\left(-\frac{\delta^2\mathbf{E}[N]}{3k}\right), \tag{16}$$

$$\Pr\left[\left|D - \mathbf{E}[D]\right| \geq \delta\mathbf{E}[D]\right] \;\leq\; 3k\exp\left(-\frac{\delta^2\mathbf{E}[D]}{3k}\right), \tag{17}$$

$$\Pr\left[\left|R - \mathbf{E}[R]\right| \geq \delta\mathbf{E}[R]\right] \;\leq\; 3k\exp\left(-\frac{\delta^2\mathbf{E}[N]}{3k}\right), \tag{18}$$

$$\Pr\left[\left|Q - \mathbf{E}[Q]\right| \geq \delta\mathbf{E}[Q]\right] \;\leq\; 3k\exp\left(-\frac{\delta^2\mathbf{E}[N]}{3k}\right) + 3k\exp\left(-\frac{\delta^2\mathbf{E}[D]}{3k}\right), \tag{19}$$

$$\Pr\left[\left|T - \mathbf{E}[T]\right| \geq \delta\mathbf{E}[T]\right] \;\leq\; 3k\exp\left(-\frac{\delta^2\mathbf{E}[N]}{3k}\right) + 3k\exp\left(-\frac{\delta^2\mathbf{E}[D]}{3k}\right). \tag{20}$$

bioRxiv preprint doi: https://doi.org/10.1101/2025.05.14.653858; this version posted July 27, 2025. The copyright holder for this preprint (which was not certified by peer review) is the author/funder, who has granted bioRxiv a license to display the preprint in perpetuity. It is made available under aCC-BY 4.0 International license.



**Proof.** *Proof of* (16)*:* Recall from the proof of Lemma 1 that we can express $N = \sum_{i=1}^{L_0} N_i$ where $N_i$ is the indicator that $K_i$ has no mutations. The $N_i$'s are not independent, but $N_i$ and $N_j$ are independent if $|i - j| \geq k$. Then, for $\ell \in \{1, \ldots, k\}$, define

$$M_\ell \triangleq \sum_{j\,:\,1 \leq \ell + kj \leq L-k+1} N_{\ell+kj}.$$

Observe that the $M_\ell$'s are defined such that each is a sum of independent identically distributed Bernoulli random variables with success probability $q = (1 - p_s - p_d)^k/(g + 1)^{k-1}$ and $N = \sum_{i=1}^{k} M_\ell$. The Chernoff bound in (23) implies that for any $\delta \in (0, 1)$,

$$\Pr\left[\left|M_\ell - \mathbf{E}[M_\ell]\right| \geq \delta \mathbf{E}[M_\ell]\right] \leq 2 \exp\left(-\frac{\delta^2 \mathbf{E}[M_\ell]}{3}\right),$$

Using the fact that $N = \sum_{i=1}^{k} M_\ell$ and Lemma 12, we get

$$\begin{aligned}
\Pr\left[\left|N - \mathbf{E}[N]\right| \geq \delta \mathbf{E}[N]\right] &= \Pr\left[\left|\sum M_\ell - \mathbf{E}[M_\ell]\right| \geq \sum \delta \mathbf{E}[M_\ell]\right] \\
&\leq \sum \Pr\left[|M_\ell - \mathbf{E}[M_\ell]| \geq \delta \mathbf{E}[M_\ell]\right] \\
&\leq \sum 2 \exp\left(-\frac{\delta^2 \mathbf{E}[M_\ell]}{3}\right), \\
&\leq 2k \exp\left(-\frac{\delta^2 \min_\ell \mathbf{E}[M_\ell]}{3}\right),
\end{aligned}$$

Finally, observe that each $M_\ell$ is the sum of at least $\lfloor \frac{L_0}{k} \rfloor$ Bernoulli variables. Then $\mathbf{E}[M_\ell] \geq \lfloor \frac{L_0}{k} \rfloor q \geq \frac{\mathbf{E}[N]}{k} - q$. Using the fact $e^{q\delta^2/(3k)} \leq e^{1/3} \leq 1.5$, we get

$$\Pr\left[\left|N - \mathbf{E}[N]\right| \geq \delta \mathbf{E}[N]\right] \leq 2k \exp\left(-\frac{\delta^2(\mathbf{E}[N] - q)}{3k}\right) \leq 3k \exp\left(-\frac{\delta^2 \mathbf{E}[N]}{3k}\right).$$

*Proof of* (17)*:* The concentration of $D$ is proven in an identical manner to the concentration of $N$. Recall from the proof of Lemma 1 that we can express $D = \sum_{i=1}^{L_0} D_i$, where $D_i$ is the indicator that the $K_i$ has no mutations except exactly one deletion. These $D_i$s have the same dependence structure as the $N_i$s, so the proof for the concentration of $D$ is identical to the one for $N$.

*Proof of* (18)*:* Since we assumed $4c_A < L$, we can multiply both sides of the inequality inside the probability of (16) by $k(L - 4c_A)$ to obtain (18).

*Proof of* (19)*:* The concentration from $Q$ comes by using Lemma 12 to combine the the concentration inequalities of $N$ and $D$. That is,

$$\begin{aligned}
\Pr\left[|Q - \mathbf{E}[Q]| \geq \delta \mathbf{E}[Q]\right] &= \Pr\left[|kN + D - k\mathbf{E}[N] - \mathbf{E}[D]| \geq \delta k \mathbf{E}[N] + \delta \mathbf{E}[D]\right] \\
&\leq \Pr\left[|kN - k\mathbf{E}[N]| \geq \delta k \mathbf{E}[N]\right] + \Pr\left[|D - \mathbf{E}[D]| \geq \delta \mathbf{E}[D]\right] \\
&= \Pr\left[|N - \mathbf{E}[N]| \geq \delta \mathbf{E}[N]\right] + \Pr\left[|D - \mathbf{E}[D]| \geq \delta \mathbf{E}[D]\right] \\
&\leq 3k \exp\left(-\frac{\delta^2 \mathbf{E}[N]}{3}\right) + 3k \exp\left(-\frac{\delta^2 \mathbf{E}[D]}{3}\right).
\end{aligned}$$

*Proof of* (20)*:* The concentration of $T$ is derived in a way identical to the concentration of $Q$. ◀

▶ **Lemma 5.** *Let* $\theta_1 = \min\left(\frac{g^2}{2(g+1)^2}, \frac{p_d}{3}\right)$*. For any* $\delta \in (0, 1)$*:*

$$\Pr\left[|L' - \mathbf{E}[L']| \geq \delta(Lg + Lp_d)\right] \leq 4e^{-\theta_1 L \delta^2}.$$

bioRxiv preprint doi: https://doi.org/10.1101/2025.05.14.653858; this version posted July 27, 2025. The copyright holder for this preprint (which was not certified by peer review) is the author/funder, who has granted bioRxiv a license to display the preprint in perpetuity. It is made available under aCC-BY 4.0 International license.



**Proof.** Let $D_i$ be an indicator that is 1 in the event that the operation at $i$ was Del. Let us define $\mathcal{L}_{\text{ins}} \triangleq \sum_{i=1}^{L} I_i$, and $\mathcal{L}_{\text{del}} \triangleq \sum_{i=1}^{L} D_i$, so that $L' = L + \mathcal{L}_{\text{ins}} - \mathcal{L}_{\text{del}}$. We will obtain the desired concentration inequality for $L'$ from separate concentration inequalities for $\mathcal{L}_{\text{ins}}$ and $\mathcal{L}_{\text{del}}$.

First, notice that $\mathcal{L}_{\text{ins}} \geq (1+\delta)Lg$ is equivalent to observing at least $(1+\delta)Lg$ failures before observing $L$ successes from a series of independent Bernoulli trials with success probability $1/(g+1)$. This is in turn equivalent to observing at most $L$ successes in $(1+\delta)Lg + L$ independent Bernoulli trials with success probability $1/(g+1)$ each. Hence, if $\{Z_i\}_{i\geq 1}$ is a sequence of independent Bernoulli random variable with success probability $1/(g+1)$, we have

$$\Pr[\mathcal{L}_{\text{ins}} \geq (1+\delta)Lg] = \Pr\Big[\sum_{i=1}^{(1+\delta)Lg+L} Z_i \leq L\Big] = \Pr\Big[\sum_{i=1}^{(1+\delta)Lg+L} Z_i \leq (1-\varepsilon)\mu\Big]$$

where

$$\mu = \mathbf{E}\Big[\sum_{i=1}^{(1+\delta)Lg+L} Z_i\Big] = \frac{(1+\delta)Lg+L}{g+1}$$

and $\varepsilon = \frac{\delta g}{\delta g+g+1}$. From the Chernoff bound in (22) we obtain

$$\Pr[\mathcal{L}_{\text{ins}} \geq (1+\delta)Lg] \leq \exp\Big(-\frac{L\delta^2 g^2}{2(g+1+\delta g)(g+1)}\Big).$$

Proceeding in an analogous manner and using the Chernoff bound of (21), we can prove the probability for the other tail

$$\Pr[\mathcal{L}_{\text{ins}} \leq (1-\delta)Lg] \leq \exp\Big(-\frac{L\delta^2 g^2}{(2+\varepsilon')(g+1-\delta g)(g+1)}\Big),$$

where $\varepsilon' = \frac{\delta g}{g+1-\delta g}$. From a union bound we deduce that:

$$\Pr[|\mathcal{L}_{\text{ins}} - Lg| \geq \delta Lg] \leq 2\exp\Big(-\frac{L\delta^2 g^2}{2(g+1-\delta g)(g+1)}\Big). \tag{25}$$

To establish the concentration of $\mathcal{L}_{\text{del}}$, note that this random variable is a sum of $L$ independent Bernoulli random variable with parameter $p_d$. Then, $\mathbf{E}[\mathcal{L}_{\text{del}}] = Lp_d$ and the Chernoff bound in (23) yields that:

$$\Pr[|\mathcal{L}_{\text{del}} - Lp_d| \geq \delta Lp_d] \leq 2\exp\Big(-\frac{Lp_d\delta^2}{3}\Big). \tag{26}$$

Using Lemma 12, we now combine the concentration bounds of (25) and (26) into a concentration bound for $L'$:

$$\begin{aligned}\Pr\Big[\big|L' - \mathbf{E}[L']\big| \geq \delta(Lg+Lp_d)\Big] &= \Pr\Big[\big|\mathcal{L}_{\text{ins}} - \mathcal{L}_{\text{del}} + Lp_d - Lg\big| \geq \delta(Lg+Lp_d)\Big] \\ &\leq \Pr[|\mathcal{L}_{\text{ins}} - Lg| \geq \delta Lg] + \Pr[|\mathcal{L}_{\text{del}} - Lp_d| \geq \delta Lp_d] \\ &\leq 2\exp\Big(-\frac{L\delta^2 g^2}{2(g+1-\delta g)(g+1)}\Big) + 2\exp\Big(-\frac{Lp_d\delta^2}{3}\Big) \\ &\leq 2\exp\Big(-\frac{L\delta^2 g^2}{2(g+1)^2}\Big) + 2\exp\Big(-\frac{Lp_d\delta^2}{3}\Big).\end{aligned}$$

Setting $\theta_1 = \min\Big(\frac{g^2}{2(g+1)^2}, \frac{p_d}{3}\Big)$, we obtain:

$$\Pr\Big[\big|L' - \mathbf{E}[L']\big| \geq \delta(Lg+Lp_d)\Big] \leq 4e^{-\theta_1\delta^2 L}.$$

◀

bioRxiv preprint doi: https://doi.org/10.1101/2025.05.14.653858; this version posted July 27, 2025. The copyright holder for this preprint (which was not certified by peer review) is the author/funder, who has granted bioRxiv a license to display the preprint in perpetuity. It is made available under aCC-BY 4.0 International license.



▶ **Lemma 6.** *Let* $\theta_2 = \max(g+1, 8)$. *Then, the following holds for any* $\delta > 0$*:*

$$\Pr\left[|P - \mathbf{E}[P]| \geq 2\delta\right] \leq 4\exp\left(-\frac{\delta^2}{8Le^{2(4\theta_2+1)}}\right) + 2\exp\left(-\frac{\delta}{2e^{4\theta_2+1}}\right).$$

*If we also assume that* $4c_A < L$, $\frac{4}{3}p_s + p_d < 1$, *and for any* $\delta_0 \in (0,1)$ *then*

$$\Pr\left[|P - \mathbf{E}[P]| \geq \delta_0 \mathbf{E}[P]\right] \leq 6\exp\left(-\frac{\delta_0^2 \mathbf{E}[P]^2}{72Le^{2(4\theta_2+1)}}\right).$$

**Proof.** We will consider the contributions to $P = L' - 4c_A{}'$ from the characters in $S$ and from newly inserted characters separately. For this, we define the random variable $X_i$ that is 0 if $S_i$ is deleted, it is $-3$ if $S_i$ is not deleted and is an 'A' after the mutation process, and it is 1 otherwise.

We also define the random variable $Z_i = I_i - 4C_i$ where recall that $I_i$ is the length of the inserted string at position $i$ and $C_i$ is the random variable corresponding to the number of 'A's in the inserted string at position $i$. Note that each inserted character contributes 1 to $P$, if it is a non-'A', or contributes $-3$ to $P$, if it is a 'A'. We can now write $P = \sum_{i=1}^{L} X_i + \sum_{i=1}^{L} Z_i$. For ease of notation, let $X = \sum_{i=1}^{L} X_i$ and $Z = \sum_{i=1}^{L} Z_i$.

Observe that the values that $X_i$ can take on are limited to the range of $[-3, 1]$. Using this and the fact that the $X_i$s are independent of each other, we apply Hoeffding's inequality (Lemma 9) to get that for any $\delta > 0$,

$$\Pr\left[|X - \mathbf{E}[X]| \geq \delta\right] \leq 2\exp\left(-\frac{\delta^2}{8L}\right). \tag{27}$$

To show that $Z$ is strongly concentrated around its mean, we first prove that the $Z_i$'s are sub-exponential random variables and then use Bernstein's inequality (Lemma 11).

▶ **Lemma 13.** *Let* $\theta_2 = \max(g+1, 8)$. *For each* $Z_i$, $\mathbf{E}[Z_i] = 0$ *and, for all* $t \geq 0$*:*

$$\Pr[|Z_i| \geq t] \leq 4e^{-t/\theta_2}.$$

We prove this lemma shortly, but first we finish the proof of Lemma 6. As the $Z_i$'s are mean-zero $(4, \theta_2)$-sub-exponential random variables, it follows from Lemma 11 that for any $\delta > 0$,

$$\Pr\left[|Z| \geq \delta\right] \leq 2\exp\left(-\min\left(\frac{\delta^2}{8Le^{2(4\theta_2+1)}}, \frac{\delta}{2e^{4\theta_2+1}}\right)\right). \tag{28}$$

We now use Lemma 12 to combine the concentrations of $X$ and $Z$ into a concentration for $P$. For any $\delta > 0$,

$$\begin{aligned}
\Pr\left[|P - \mathbf{E}[P]| \geq 2\delta\right] &= \Pr\left[|X - \mathbf{E}[X] + Z - \mathbf{E}[Z]| \geq 2\delta\right] \\
&\leq \Pr\left[|X - \mathbf{E}[X]| \geq \delta\right] + \Pr\left[|Z - \mathbf{E}[Z]| \geq \delta\right] \\
&\leq 2\exp\left(-\frac{\delta^2}{8L}\right) + 2\exp\left(-\min\left(\frac{\delta^2}{8Le^{2(4\theta_2+1)}}, \frac{\delta}{2e^{4\theta_2+1}}\right)\right) \\
&\leq 2\exp\left(-\frac{\delta^2}{8L}\right) + 2\exp\left(-\frac{\delta^2}{8Le^{2(4\theta_2+1)}}\right) + 2\exp\left(-\frac{\delta}{2e^{4\theta_2+1}}\right) \\
&\leq 4\exp\left(-\frac{\delta^2}{8Le^{2(4\theta_2+1)}}\right) + 2\exp\left(-\frac{\delta}{2e^{4\theta_2+1}}\right).
\end{aligned}$$

bioRxiv preprint doi: https://doi.org/10.1101/2025.05.14.653858; this version posted July 27, 2025. The copyright holder for this preprint (which was not certified by peer review) is the author/funder, who has granted bioRxiv a license to display the preprint in perpetuity. It is made available under aCC-BY 4.0 International license.



Further, applying the assumptions that $\frac{4}{3}p_s + p_d < 1$ and $4c_A < L$ to the expected value of $P$ given by (5), we have that $0 < \mathbf{E}[P] < L$. Therefore, given $\delta_0 \in (0,1)$, we can plug $\delta = \delta_0\mathbf{E}[P]/2$ into the above to obtain

$$\Pr\Big[|P - \mathbf{E}[P]| \geq \delta_0\mathbf{E}[P]\Big] \leq 4\exp\left(-\frac{\delta_0^2\mathbf{E}[P]^2}{32Le^{2(4\theta_2+1)}}\right) + 2\exp\left(-\frac{\delta_0\mathbf{E}[P]}{4e^{4\theta_2+1}}\right) \leq 6\exp\left(-\frac{\delta_0^2\mathbf{E}[P]^2}{32Le^{2(4\theta_2+1)}}\right).$$

◀

**Proof of Lemma 13.** Recall that $Z_i = I_i - 4C_i$, where $I_i$ is a Geometric random variable with parameter $p \triangleq 1/(g+1)$ and mean $g$, and $C_i$ is a Binomial random variable with parameters $I_i$ and $1/4$. Observe that $\mathbf{E}[Z_i] = 0$, since $\mathbf{E}[I_i] = g$ and, by (24), $\mathbf{E}[C_i] = g/4$. Let $B_y$ be a Binomial random variable with parameters $y$ and $1/4$. For $t \geq 0$, conditioning on the value of $I_i$ and using Hoeffding's inequality (Lemma 9), we obtain:

$$\begin{aligned}
\Pr[|Z_i| \geq t] &= \sum_{y\geq 0} \Pr[I_i = y]\Pr[|B_y - y/4| \geq t/4] \\
&\leq 2p\sum_{y\geq 0}(1-p)^y \exp\Big(\frac{-t^2}{8y}\Big) \\
&\leq 2p\left(\sum_{y=0}^{\lfloor t\rfloor}(1-p)^y\exp\Big(-\frac{t^2}{8y}\Big) + \sum_{y=\lfloor t\rfloor+1}^{\infty}(1-p)^y\exp\Big(-\frac{t^2}{8y}\Big)\right).
\end{aligned}$$

For $y \leq t$, we have $\frac{t^2}{8y} \geq \frac{t}{8}$, so $\exp(-\frac{t^2}{8y}) \leq \exp(-t/8)$ and we have

$$\sum_{y=0}^{\lfloor t\rfloor}(1-p)^y\exp(-\frac{t^2}{8y}) \leq \exp(-t/8)\sum_{y=0}^{\lfloor t\rfloor}(1-p)^y \leq \frac{e^{-t/8}}{p}.$$

When $y > t$, we use the fact that $e^{-x} \leq 1$ for all positive $x$ and apply the formula for a geometric series; we have

$$\sum_{y=\lfloor t\rfloor+1}^{\infty}(1-p)^y\exp(-\frac{t^2}{8y}) \leq \sum_{y=\lfloor t\rfloor+1}^{\infty}(1-p)^y \leq \frac{(1-p)^t}{p} \leq \frac{e^{-pt}}{p}.$$

Putting these bounds together:

$$\Pr[|Z_i| \geq t] \leq 2\left(e^{-t/8} + e^{-pt}\right) \leq 4e^{-t/\theta_2},$$

where $\theta_2 = \min\{p, 1/8\} = \max(g+1, 8)$. ◀

▶ **Lemma 7.** *Let $X$, $Y$, and $Z$ be random variables with strictly positive means. Let $\delta \in (0, 1/5)$. Suppose that $(1-\delta)\,\mathbf{E}[X] \leq X \leq (1+\delta)\,\mathbf{E}[X]$ and $(1-\delta)\,\mathbf{E}[Y] \leq Y \leq (1+\delta)\,\mathbf{E}[Y]$. Then*

$$(1-4\delta)\frac{\mathbf{E}[X]}{\mathbf{E}[Y]} \leq \frac{X}{Y} \leq (1+4\delta)\frac{\mathbf{E}[X]}{\mathbf{E}[Y]}.$$

*If it also holds that $(1-\delta)\,\mathbf{E}[Z] \leq Z \leq (1+\delta)\,\mathbf{E}[Z]$, then*

$$(1-4\delta)\frac{\mathbf{E}[X]\mathbf{E}[Y]}{\mathbf{E}[Z]} \leq \frac{XY}{Z} \leq (1+4\delta)\frac{\mathbf{E}[X]\mathbf{E}[Y]}{\mathbf{E}[Z]},$$

**Proof.** First, using the fact that $\frac{(1+\delta)^2}{1-\delta} \leq 1+4\delta$, we get:

$$\frac{XY}{Z} \leq \frac{(1+\delta)^2}{1-\delta}\frac{\mathbf{E}[X]\mathbf{E}[Y]}{\mathbf{E}[Z]} \leq (1+4\delta)\frac{\mathbf{E}[P]\mathbf{E}[Q]}{\mathbf{E}[T]}.$$

bioRxiv preprint doi: https://doi.org/10.1101/2025.05.14.653858; this version posted July 27, 2025. The copyright holder for this preprint (which was not certified by peer review) is the author/funder, who has granted bioRxiv a license to display the preprint in perpetuity. It is made available under aCC-BY 4.0 International license.



Using the fact that $\frac{(1-\delta)^2}{1+\delta} \geq 1 - 4\delta$, we get

$$\frac{XY}{Z} \geq \frac{(1-\delta)^2}{1+\delta}\frac{\mathbf{E}[X]\mathbf{E}[Y]}{\mathbf{E}[Z]} \geq (1-4\delta)\frac{\mathbf{E}[X]\mathbf{E}[Y]}{\mathbf{E}[Z]}$$

Similarly, using the facts that $\frac{1-\delta}{1+\delta} \geq 1 - 3\delta$ and $\frac{1+\delta}{1-\delta} \leq 1 + 3\delta$, we obtain that $(1-3\delta)\frac{\mathbf{E}[X]}{\mathbf{E}[Y]} \leq \frac{X}{Y} \leq (1+3\delta)\frac{\mathbf{E}[X]}{\mathbf{E}[Y]}$. ◀

▶ **Theorem 2.** *Let* $\theta_1 = \min\left(\frac{g^2}{2(g+1)^2}, \frac{p_d}{3}\right)$ *and* $\theta_2 = \max(g+1, 8)$. *Suppose* $4c_A < L$ *and* $\frac{4}{3}p_s + p_d < 1$. *Then, for* $\delta \in (0, 1/5)$*:*

1. $$\Pr[|\hat{p}_d - p_d| \geq 4\delta] \leq 6k\exp\left(-\frac{\delta^2\mathbf{E}[D]}{3k}\right) + 3k\exp\left(-\frac{\delta^2\mathbf{E}[N]}{3k}\right) + 6\exp\left(-\frac{\delta^2\mathbf{E}[P]^2}{72Le^{2(4\theta_2+1)}}\right).$$

2. $$\Pr[|\hat{p}_s - p_s| \geq 12\delta] \leq 9k\exp\left(-\frac{\delta^2\mathbf{E}[N]}{3k}\right) + 6k\exp\left(-\frac{\delta^2\mathbf{E}[D]}{3k}\right) + 6\exp\left(-\frac{\delta^2\mathbf{E}[P]^2}{72Le^{2(4\theta_2+1)}}\right).$$

3. $$\Pr[|\hat{g}-g| \geq (5+g)\delta] \leq 6k\exp\left(-\frac{\delta^2\mathbf{E}[D]}{3k}\right)+3k\exp\left(-\frac{\delta^2\mathbf{E}[N]}{3k}\right)+6\exp\left(-\frac{\delta^2\mathbf{E}[P]^2}{72Le^{2(4\theta_2+1)}}\right)+4\exp\left(-\theta_1 L\delta^2\right).$$

**Proof.** In Section 4, we proved the concentration bound for $\hat{p}_d$; here, we will prove the bound for $\hat{p}_s$ and $g$. Recall that $P = L' - 4c'_A$, $Q = kN + D$, $R = k(L - 4c_A)N$, and $T = 4kN + D$. Let us assume our variables are indeed close to their means, i.e. for all $X \in \{P, Q, R, T\}$, $(1-\delta)\,\mathbf{E}[X] \leq X \leq (1+\delta)\,\mathbf{E}[X]$ for $\delta \in (1, 1/5)$. Because we assume that $\frac{4}{3}p_s + p_d < 1$ and $4c_A < L$, we use (5) to obtain that $\mathbf{E}[P] > 0$; in addition, Lemma 1 gives $\mathbf{E}[Q], \mathbf{E}[R], \mathbf{E}[T] > 0$. Therefore, we can apply Lemma 7 to get

$$\frac{PQ - R}{T} \leq (1+4\delta)\frac{\mathbf{E}[P]\mathbf{E}[Q]}{\mathbf{E}[T]} - (1-4\delta)\frac{\mathbf{E}[R]}{\mathbf{E}[T]} \quad \leq \frac{\mathbf{E}[P]\mathbf{E}[Q] - \mathbf{E}[R]}{\mathbf{E}[T]} + 4\delta\frac{\mathbf{E}[P]\mathbf{E}[Q] + \mathbf{E}[R]}{\mathbf{E}[T]}$$

and, similarly, $\frac{PQ-R}{T} \geq \frac{\mathbf{E}[P]\mathbf{E}[Q]-\mathbf{E}[R]}{\mathbf{E}[T]} - 4\delta\frac{\mathbf{E}[P]\mathbf{E}[Q]+\mathbf{E}[R]}{\mathbf{E}[T]}$. Multiplying by $\frac{-3}{L-4c_A}$ and using the definition of (13), we obtain that $p_s - 4\delta W \leq \hat{p}_s \leq p_s + 4\delta W$ with $W = 3\frac{\mathbf{E}[P]\mathbf{E}[Q]+\mathbf{E}[R]}{(L-4c_A)\mathbf{E}[T]}$. Plugging in the values given by Lemma 1, we obtain that $W \leq 3$. Hence, $|\hat{p}_s - p_s| \leq 12\delta$. As in the proof for $\hat{p}_d$, the result follows by a union bound using the individual concentration bounds for $P$ (Lemma 6), $Q$, $R$, and $T$ (Lemma 4).

To obtain the bound for $\hat{g}$, recall that $\hat{g} = \frac{L'}{L} - 1 + \hat{p}_d$. From Lemma 5, for any $\delta \in (0, 1)$

$$\Pr[|L' - \mathbf{E}[L']| \geq \delta(g + p_d)L] \leq 4e^{-\theta_1 L\delta^2},$$

where $\theta_1 = \min\left(\frac{g^2}{2(g+1)^2}, \frac{p_d}{3}\right)$. Then,

$$\Pr\left[\left|\frac{L'}{L} - \frac{\mathbf{E}[L']}{L}\right| \geq \delta(g+1)\right] \leq \Pr\left[\left|\frac{L'}{L} - \frac{\mathbf{E}[L']}{L}\right| \geq \delta(g + p_d)\right] \leq 4e^{-\theta_1 L\delta^2}.$$

Combining this with Lemma 12, we get

$$\begin{aligned}\Pr[|\hat{g} - g| \geq (5+g)\delta] &= \Pr\left[\left|\frac{L'}{L} - \frac{\mathbf{E}[L']}{L} + \hat{p}_d - p_d\right| \geq (5+g)\delta\right] \\ &\leq \Pr\left[\left|\frac{L'}{L} - \frac{\mathbf{E}[L']}{L}\right| \geq (1+g)\delta\right] + \Pr\left[|\hat{p}_d - p_d| \geq 4\delta\right] \\ &\leq 4e^{-\theta_1 L\delta^2} + \Pr\left[|\hat{p}_d - p_d| \geq 4\delta\right].\end{aligned}$$

Plugging in the bounds on $\Pr\left[|\hat{p}_d - p_d| \geq 4\delta\right]$ from part 2 of this theorem gives the result. ◀

bioRxiv preprint doi: https://doi.org/10.1101/2025.05.14.653858; this version posted July 27, 2025. The copyright holder for this preprint (which was not certified by peer review) is the author/funder, who has granted bioRxiv a license to display the preprint in perpetuity. It is made available under aCC-BY 4.0 International license.



## C Proofs of helper lemmas

In this section, we provide for completeness some of the proofs from Appendix A. To prove Lemma 11, we will first need the following fact.

▶ **Lemma 14.** *Let $a, b$ be fixed positive real numbers. If $X$ is $(a, b)$-sub-exponential, for $\lambda \in \mathbb{R}$ such that $|\lambda e^{ab+1}| < 1/2$ we have $\mathbf{E}[e^{\lambda X}] \le e^{2(\lambda e^{a+1}b)^2}$.*

**Proof.** Using the Taylor expansion for the exponential function, the fact that $\mathbf{E}[X] = 0$, and standard inequality $p! \ge (\frac{p}{e})^p$, we deduce that

$$\mathbf{E}[e^{\lambda X}] = 1 + \lambda \mathbf{E}[X] + \sum_{p=2}^{\infty} \frac{\lambda^p \mathbf{E}[|X|^p]}{p!} = 1 + \sum_{p=2}^{\infty} \left(\frac{\lambda e}{p}\right)^p \mathbf{E}[|X|^p]. \tag{29}$$

Observe that for integer $p \ge 1$, we have the identity $\mathbf{E}[|X|^p] = \int_0^\infty \Pr[|X|^p \ge u]du$, and with the change of variable $u = bt^p$, we obtain:

$$\mathbf{E}[|X|^p] = \int_0^\infty \Pr[|X|^p \ge bt^p](bp)t^{p-1}dt \le abp \int_0^\infty e^{-t}t^{p-1}du = abp\Gamma(p) \le abpp^p \le (e^{ab}p)^p;$$

$\Gamma$ denotes the Gamma function and we use the fact that $\Gamma(x) \le x^x$ for all $x \ge 1$. Plugging this bound into (29), we obtain:

$$\mathbf{E}[e^{\lambda X}] \le 1 + \sum_{p=2}^{\infty} (\lambda e^{ab+1})^p = 1 + \frac{(\lambda e^{ab+1})^2}{1 - \lambda e^{ab+1}} \le 1 + 2(\lambda e^{ab+1})^2 \le e^{2(\lambda e^{ab+1})^2}$$

provided $|\lambda e^{ab+1}| < 1/2$. ◀

**Proof of Lemma 11.** Let $\lambda > 0$. Markov's inequality and the independence of the $X_i$s imply that:

$$\Pr[X \ge t] = \Pr\left[e^{\lambda X} \ge e^{\lambda t}\right] \le e^{-\lambda t}\mathbf{E}[e^{\lambda X}] = e^{-\lambda t} \prod_{i=1}^{n} \mathbf{E}[e^{\lambda X_i}].$$

If $\lambda$ is such that $|\lambda e^{ab+1}| < 1/2$, Lemma 14 implies that $\mathbf{E}[e^{\lambda X_i}] \le e^{2(\lambda e^{ab+1})^2}$ and thus

$$\Pr[X \ge t] \le e^{-\lambda t + 2n(\lambda e^{ab+1})^2}.$$

For ease of notation set $y = e^{ab+1}$, and let us optimize for $\lambda$. The minimum of the parabola $f(\lambda) = -\lambda t + 2y^2 n \lambda^2$ in $[0, 1/(2y)]$ aligns with is global minimum at $\lambda = \frac{t}{4y^2 n}$ when $\frac{t}{4y^2 n} \le \frac{1}{2y}$; the minimum value of $f$ is $\frac{t^2}{8ny^2}$ in this case. Otherwise, if $\frac{t}{4y^2 n} > \frac{1}{2y}$, the the minimum occurs at the boundary point $\frac{1}{2y}$ and has value $\frac{-t}{2y} + \frac{n}{2} \ge \frac{-t}{2y}$. Combining these two bounds:

$$\Pr[X \ge t] \le e^{-\min\left(\frac{t^2}{8ne^{2(ab+1)}}, \frac{t}{2e^{ab+1}}\right)}.$$

Repeating the same argument, we obtain the same bound for $\Pr[-X \ge t]$ and the result follows from a union bound. ◀

**Proof of Lemma 12.** First observe that $|\sum X_i| \le \sum |X_i|$ and therefore $|\sum X_i| \ge \sum t_i$ implies that $\sum |X_i| \ge \sum t_i$. Hence $\Pr[|\sum X_i| \ge \sum t_i] \le \Pr[\sum |X_i| \ge \sum t_i]$. Second, $\sum |X_i| \ge \sum t_i$ implies that there is at least one $i$ such that $|X_i| \ge t_i$. Therefore, $\Pr[\sum |X_i| \ge \sum t_i] \le \Pr[|X_1| \ge t_1 \cup \ldots \cup |X_n| \ge t_n]$. Third, by the union bound, $\Pr[|X_1| \ge t_1 \cup \ldots \cup |X_n| \ge t_n] \le \sum \Pr[|X_i| \ge t_i]$. Combining the three observations in sequences, we get the result. ◀
