# Supplementary material for: Estimation of substitution and indel rates via k-mer statistics
Source: bioRxiv. 2025 May 18:2025.05.14.653858. Preprint. [Version 2] doi: 10.1101/2025.05.14.653858 (PMC12132262; doi:10.1101/2025.05.14.653858)
Supplement: Supplementary file 1 [file NIHPP2025.05.14.653858v2-supplement-1.pdf]

bioRxiv preprint doi: https://doi.org/10.1101/2025.05.14.653858; this version posted May 18, 2025. The copyright holder for this preprint (which was not certified by peer review) is the author/funder, who has granted bioRxiv a license to display the preprint in perpetuity. It is made available under aCC-BY 4.0 International license.

Supplementary Materials

The full version of this manuscript, including proofs in an extended abstract, is available on the BioRxiv: https://tinyurl.com/5d8fw2y2.

A List of notations used in the manuscript

In this section, we list all the notations used in the manuscript.

Table 1 List of notations and their meanings

Notation	Meaning
**E**	Expectation
**Var**	Variance
Pr	Probability of an event
inf	Infimum
exp	Exponent to e
min	Minimum
max	Maximum
p_s	Substitution rate
p_d	Deletion rate
d	Average insertion length
S	The original string
S'	The mutated string
L	Length of (number of characters in) S
L'	Length of (number of characters in) S'
f_A, f_C, f_G, f_T	Number of 'A', 'C', 'G', 'T' in S, respectively. Here, $f_A + f_C + f_G + f_T = L$.
$f_A{}', f_C{}', f_G{}', f_T{}'$	Number of 'A', 'C', 'G', 'T' in S', respectively. Here, $f_A{}' + f_C{}' + f_G{}' + f_T{}' = L'$.
P	$P = L' - 4f_A{}'$
$\mathcal{K}$	Number of k-mers in S. Here, $\mathcal{K} = L - k + 1$.
$\mathcal{K}'$	Number of k-mers in S'. Here, $\mathcal{K}' = L' - k + 1$.
i	A position in the string
S_i	The character in S at position i
$Q^{(i)}$	The string inserted after S_i, by the mutation process
l_i	Length of $Q^{(i)}$
D_i	Indicator that S_i was deleted by the mutation process
A_i	Indicator that stay action was chosen at position i
B_i	Indicator that S'_i = 'A', where $S_i \neq$ 'A'
C_i	Number of 'A's in $Q^{(i)}$
$\mathcal{K}_i$	The k-mer in S starting at position i, ending at position $i + k - 1$
N_i	Indicator that the mutation process did not mutate $\mathcal{K}_i$
E_i	Indicator that a single substitution, and no other mutation was introduced in $\mathcal{K}_i$
Δ_i	Indicator that a single deletion, and no other mutation was introduced in $\mathcal{K}_i$
$\mathcal{N}$	Number of k-mers that were not mutated by the mutation process
$\mathcal{S}$	Number of k-mers where a single substitution and no other mutation was introduced by the mutation process
$\mathcal{D}$	Number of k-mers where a single deletion and no other mutation was introduced by the mutation process

bioRxiv preprint doi: https://doi.org/10.1101/2025.05.14.653858; this version posted May 18, 2025. The copyright holder for this preprint (which was not certified by peer review) is the author/funder, who has granted bioRxiv a license to display the preprint in perpetuity. It is made available under aCC-BY 4.0 International license.

Notation	Meaning
$\mathcal{I}$	Number of k-mers where a single insertion and no other mutation was introduced by the mutation process
$\mathcal{L}_{\text{ins}}$	Total number of characters inserted by the mutation process.
$\mathcal{L}_{\text{del}}$	Total number of characters deleted by the mutation process.
$\mathcal{A}$	$\sum_{i:S_i=\text{'A'}} A_i$
$\mathcal{B}$	$\sum_{i:S_i\neq\text{'A'}} B_i$
C	$\sum_{i=1}^{L} C_i$
M_i	$\sum_{j=0}^{c-1} N_{i+kj}$, where $\mathcal{K} = ck$ (assuming $\mathcal{K}$ is a multiple of k)

B Preliminaries

This section gathers several definitions and standard concentration inequalities that we use in our proofs. For a random variable X with finite non-zero variance σ^2, Chebyshev's inequality [2] says that for any real value r

$$\Pr\left[|X - \mathbf{E}[X]| \geq r\sigma\right] \leq \frac{1}{r^2}.$$

Sums of independent random variables exhibit sharper concentration, as captured by the following standard inequalities. The first one is the Chernoff bound for sums of independent and identically distributed Bernoulli random variables [4].

▶ **Lemma 11.** *Let $X_1, \ldots, X_n$ be a sequence of independently and identically distributed Bernoulli random variables. Consider $X = \sum_{i=1}^n X_i$ and let $\mathbf{E}[X] = \mu$. Then, the following three inequalities hold:*

$$\Pr(X \geq (1+\delta)\mu) \leq e^{-\delta^2\mu/(2+\delta)}, \qquad 0 \leq \delta, \tag{17}$$

$$\Pr(X \leq (1-\delta)\mu) \leq e^{-\delta^2\mu/2}, \qquad 0 < \delta < 1, \tag{18}$$

$$\Pr(|X-\mu| \geq \delta\mu) \leq 2e^{-\delta^2\mu/3}, \qquad 0 < \delta < 1. \tag{19}$$

A more general inequality that applies to sums of independent and bounded random variables is Hoeffding's inequality [9].

▶ **Lemma 12.** *Let $X_1, \ldots, X_n$ be a sequence of independently and identically distributed random variables taking values in $[a, b]$. Let $X = \sum_{i=1}^n X_i$, and $\mathbf{E}[X] = \mu$. Then, for any $\delta > 0$:*

$$\Pr\left(|X-\mu| \geq \delta\right) \leq 2e^{-\frac{2\delta^2}{n(b-a)^2}}. \tag{20}$$

We will analyze sums of *unbounded* random variables. For this, we recall the definition of sub-exponential random variables. For a comprehensive survey about this type of random variables, the reader is referred to [20, Chapter 2.7].

▶ **Definition 13.** *A random variable X such that $\mathbf{E}[X] = 0$ is called sub-exponential if either of the following equivalent properties holds:*

1. *There exists $K_1 > 0$ such that for all $t \geq 0$:*

$$\Pr\left[|X| \geq t\right] \leq 2\exp\{-t/K_1\}.$$

2. *There exists $K_2 > 0$ such that*

$$\mathbf{E}\left[e^{|X|/K_2}\right] \leq 2. \tag{21}$$

bioRxiv preprint doi: https://doi.org/10.1101/2025.05.14.653858; this version posted May 18, 2025. The copyright holder for this preprint (which was not certified by peer review) is the author/funder, who has granted bioRxiv a license to display the preprint in perpetuity. It is made available under aCC-BY 4.0 International license.

The constants K_1 and K_2 differ by an absolute multiplicative constant. The smallest K_2 such that Equation (21) holds is called the *sub-exponential norm of* X and is denoted by $\|X\|_{\psi_1}$.

For sums of sub-exponential random variables, the following version of Bernstein's inequality holds; see [20, Theorem 2.8.1].

▶ **Lemma 14.** *Let $X_1, \ldots, X_n$ be a sequence of independent, mean-zero, sub-exponential random variables, and let $X = \sum_{i=1}^n X_i$. Then, there exists an absolute constant $c > 0$ such that for every $t \geq 0$:*

$$\Pr[|X| \geq t] \leq 2\exp\left[-c\min\left(\frac{t^2}{\sum_{i=1}^N \|X_i\|_{\psi_1}^2}, \frac{t}{\max_i \|X_i\|_{\psi_1}}\right)\right].$$

For the reader's convenience, our notations are summarized in Table 1.

C Proofs of theorems

▶ **Lemma 1.** *Let L' be the length of S'. Then*

$$\mathbf{E}[L'] = L\,(1 + d - p_d), \tag{1}$$

$$\mathbf{Var}[L'] = L\big(d(d+1) + p_d(1-p_d)\big).$$

Proof. We note that the mutation process goes through every position in S, and removes the character S_i with probability p_d, and inserts a string $Q^{(i)}$ of length l_i. Only these two operations change the number of characters in S – substitute and stay actions do not change the number of total characters.

Let D_i be an indicator variable defined as follows:

$$D_i = \begin{cases} 1 & \text{if } S_i \text{ was removed,} \\ 0 & \text{otherwise;} \end{cases}$$

for $1 \leq i \leq L$. Using the D_i's, we can express L' as follows:

$$L' = L + \sum_{i=1}^{L} l_i - \sum_{i=1}^{L} D_i \ . \tag{22}$$

We can compute the expected length of S' by using the linearity of expectation. We note that l_i is a random variable that follows the geometric distribution with a mean of d, and therefore, $\mathbf{E}[l_i] = d$. The indicator variables D_i's take a value 1 with probability p_d, and therefore, $\mathbf{E}[D_i] = p_d$. Using these facts and the linearity of expectation, Equation (1) is proved.

We next compute the variance of L'. Note that D_i's are indicator variables, and therefore, $\mathbf{Var}[D_i] = p_d(1-p_d)$. We also observe that l_i's are geometrically distributed with mean d where $d \geq 0$, and therefore, $\mathbf{Var}[l_i] = d(d+1)$. Note that all l_i's and all D_i's are mutually independent. Using the fact that D_i's and l_i's are independent proves the variance and completes the proof. ◀

▶ **Lemma 2.** *Let f_A and $f_A{}'$ be the number of 'A's in S and S', respectively. Then,*

$$\mathbf{E}[f_A{}'] = f_A(1 - p_s - p_d) + \frac{p_s(L - f_A)}{3} + \frac{dL}{4}. \tag{2}$$

Proof. We can quantify $f_A{}'$ by introducing a few random variables.

Let A_i be an indicator defined as follows:

$$A_i = \begin{cases} 1 & \text{if } a_i \text{ was a stay operation,} \\ 0 & \text{otherwise;} \end{cases}$$

bioRxiv preprint doi: https://doi.org/10.1101/2025.05.14.653858; this version posted May 18, 2025. The copyright holder for this preprint (which was not certified by peer review) is the author/funder, who has granted bioRxiv a license to display the preprint in perpetuity. It is made available under aCC-BY 4.0 International license.

defined only for the positions i such that S_i = 'A'. We note that the probability of $A_i = 1$ is $1 - p_s - p_d$, and therefore, $\mathbf{E}[A_i] = 1 - p_s - p_d$.

Let us also define B_i as follows:

$$B_i = \begin{cases} 1 & \text{if } a_i \text{ was a substitute operation, and } S'_i = \text{'A'}, \\ 0 & \text{otherwise;} \end{cases}$$

defined only for the positions i such that $S_i \neq$ 'A'. It is straightforward to argue that the probability of $B_i = 1$ is $p_s/3$, and therefore, $\mathbf{E}[B_i] = p_s/3$.

And finally, let C_i be the number of As in $Q^{(i)}$, defined for $i = 1$ to L. Note that every character in $Q^{(i)}$ can be an 'A' with probability 1/4. Also, note that the total number of characters in $Q^{(i)}$ is l_i, which is drawn from a geometric distribution with mean d. Therefore, it is straightforward to derive that $\mathbf{E}[C_i] = d/4$.

Using these indicators, we can quantify $f_A{}'$ as follows:

$$f_A{}' = \sum_{i:S_i=\text{'A'}} A_i + \sum_{i:S_i\neq\text{'A'}} B_i + \sum_{i=1}^{L} C_i. \tag{23}$$

Plugging the expected values of A_i's, B_i's and C_i's in Equation (23) completes the proof.

◀

▶ **Lemma 3.** $\mathbf{E}[\mathcal{N}] = \mathcal{K}\,(1 - p_s - p_d)^{\,k}\,\frac{1}{(d+1)^{\,k-1}}$.

Proof. For a k-mer to have no mutation, the mutation model would have to choose the stay operation for all of its k positions, the probability of which is $(1 - p_s - p_d)^k$. Additionally, for all the $k - 1$ insert positions, the mutation model would need to choose strings of length zero. The probability of selecting 0 from a geometric distribution with mean $d \geq 0$ is $1/(d + 1)$. Therefore, the probability that 0 was sampled for all these $k - 1$ positions is $1/(d + 1)^{k-1}$.

Using these facts, we can write that the probability that a k-mer does not have a mutation is $(1 - p_s - p_d)^k/(d + 1)^{k-1}$. Let N_i be the indicator variable defined as follows:

$$N_i = \begin{cases} 1 & \text{if } k\text{-mer starting at position } i \text{ has no mutation,} \\ 0 & \text{otherwise;} \end{cases}$$

for $1 \leq i \leq \mathcal{K}$. Let $\mathcal{N}$ be the number of k-mers that have no mutation. Then, we can write the following:

$$\mathcal{N} = \sum_{i=1}^{\mathcal{K}} N_i\ . \tag{24}$$

Using the fact that $\mathbf{E}[N_i] = (1 - p_s - p_d)^k/(d + 1)^{k-1}$, we apply linearity of expectation in the above equation, which completes the proof.

◀

▶ **Lemma 4.** *The expectations of $\mathcal{S}$, $\mathcal{D}$, and $\mathcal{I}$ are given by:*

$$\mathbf{E}[\mathcal{S}] = \mathcal{K}\,k\,(1 - p_s - p_d)^{k-1}\,p_s\,\frac{1}{(d+1)^{\,k-1}}. \tag{3}$$

$$\mathbf{E}[\mathcal{D}] = \mathcal{K}\,k\,(1 - p_s - p_d)^{k-1}\,p_d\,\frac{1}{(d+1)^{\,k-1}}. \tag{4}$$

$$\mathbf{E}[\mathcal{I}] = \mathcal{K}\,(k-1)\,(1 - p_s - p_d)^{k}\,\frac{d}{(d+1)^{\,k}}. \tag{5}$$

bioRxiv preprint doi: https://doi.org/10.1101/2025.05.14.653858; this version posted May 18, 2025. The copyright holder for this preprint (which was not certified by peer review) is the author/funder, who has granted bioRxiv a license to display the preprint in perpetuity. It is made available under aCC-BY 4.0 International license.

Proof. Let E_i be an indicator random variable defined as follows for $1 \le i \le \mathcal{K}$:

$$E_i = \begin{cases} 1 & \text{if } k\text{-mer starting at position } i \text{ has a single substitution and no other mutation,} \\ 0 & \text{otherwise.} \end{cases}$$

We note that for a k-mer to have introduced with only a single substitution and no other mutations, the mutation process needs to insert 0 length strings in all the $k-1$ insert positions, and other than a single substitution position, for all other $k-1$ characters in the k-mer, the mutation process needs to select a stay operation. The single substitution operation could be any of the k positions. Using these facts, we can write that $\mathbf{E}[S_i] = k\,(1-p_s-p_d)^{k-1}p_s/(d+1)^{k-1}$. Let $\mathcal{S}$ be the number of total k-mers with a single substitution and no other mutation. Then, we have the following:

$$\mathcal{S} = \sum_{i=1}^{\mathcal{K}} E_i \; .$$

Equation (3) follows from the above using linearity of expectation. The expectations of $\mathcal{D}$ and $\mathcal{I}$ are also derived following a similar argument.

◀

▶ **Lemma 6.** *For any $\delta \in (0,1)$:*

$$\Pr\left[\left|\mathcal{N} - \mathbf{E}[\mathcal{N}]\right| \ge \delta\mathbf{E}[\mathcal{N}]\right] \;\le\; 2k\exp\left\{-\frac{\delta^2\mathbf{E}[\mathcal{N}]}{3k}\right\}.$$

Proof. Recall the definition of $\mathcal{N}$ in terms of N_i indicator random variables, introduced in the proof of Lemma 3. We showed that that $\Pr[N_i = 1] = (1-p_s-p_d)^k/(d+1)^{k-1}$. We observe that N_i's are not independent of each other. However, the dependency only exists within a window of k k-mers in each direction. In other words, N_i is only dependent on N_j' s, where $j = \max\{0, i-k+1\}$ to $\min\{i+k-1, L\}$.

For ease of exposition, we treat only the case $\mathcal{K} \equiv 0 \mod k$. When $\mathcal{K} = ck + r$ with $0 < r < k$, we either pad the sequence with $k-r$ additional positions (alternatively trim it), or group the indices by residue class modulo k allowing the last block(s) to have size $c+1$ (so then $\mathbf{E}[M_i]$ takes value of cq or $(c+1)q$). In either case, the resulting concentration inequality remains unchanged. So without the loss of generality, we assume that $\mathcal{K}$, the number of k-mers in S, is a multiple of k. That is, $\mathcal{K} = ck$, where $c \in \mathbb{N}$. Using these notations, we define a set of random variables M_i as follows:

$$M_i = \sum_{j=0}^{c-1} N_{i+kj},$$

where $i = 1, 2, 3, \cdots, k$. We defined M_i's in a way such that each M_i is a sum of c independent Bernoulli random variables, each with success probability $\Pr[N_i = 1] = (1-p_s-p_d)^k/(d+1)^{k-1}$. For notational simplicity, let $q = \Pr[N_i = 1]$.

It follows that $\mathbf{E}[\mathcal{N}] = \mathcal{K}q$. Also, the expected value of M_i is given by the following.

$$\mathbf{E}[M_i] = cq = \frac{\mathcal{K}}{k}q,$$

where $i = 1, 2, 3, \cdots, k$. Additionally, as each M_i is a sum of iid Bernoulli random variables, we can use the Chernoff inequality in Equation (19) for M_i. Doing so gives us the following result:

bioRxiv preprint doi: https://doi.org/10.1101/2025.05.14.653858; this version posted May 18, 2025. The copyright holder for this preprint (which was not certified by peer review) is the author/funder, who has granted bioRxiv a license to display the preprint in perpetuity. It is made available under aCC-BY 4.0 International license.

$$\Pr\left[\left|M_i - \frac{\mathcal{K}}{k}q\right| \geq \delta\frac{\mathcal{K}}{k}q\right] \;\leq\; 2\exp\left\{-\frac{\mathcal{K}\delta^2 q}{3k}\right\},$$

which holds for $i = 1, 2, 3, \cdots, k$. Taking a union bound of these k inequalities completes the proof. ◀

▶ **Lemma 7.** *For any* $\delta \in (0, 1)$*:*

$$\Pr\left[\left|\mathcal{D} - \mathbf{E}[\mathcal{D}]\right| \geq \delta\mathbf{E}[\mathcal{D}]\right] \;\leq\; 2k\exp\left\{-\frac{\delta^2\mathbf{E}[\mathcal{D}]}{3k}\right\}.$$

Proof. Using the same terminology used to prove Equation (4), we observe that every k-mer can be mutated with a single deletion and no other mutation with a probability of $k\left(1-p_s-p_d\right)^{k-1} p_d \frac{1}{\left(d+1\right)^{k-1}}$. Let us define the following sequence of indicator variables:

$$\Delta_i = \begin{cases} 1 & \text{if the mutation process introduces only a single deletion to the } k\text{-mer starting at position } i, \\ 0 & \text{otherwise;} \end{cases}$$

for $i = 1$ to L. We note that the following holds:

$$\mathbf{E}[\Delta_i] = \Pr[\Delta_i = 1] = k\left(1 - p_s - p_d\right)^{k-1} p_d \frac{1}{\left(d+1\right)^{k-1}},$$

for $i = 1$ to L. Using these indicator variables, we have the following:

$$\mathcal{D} = \sum_{i=1}^{L} \Delta_i.$$

We observe that Δ_i's have the same dependency nature as N_i's as we observed in the proof of Lemma 6: Δ_i only depends on Δ_j' s, where $j = \max\{0, i-k+1\}$ to $\min\{i+k-1, L\}$. Using the same k-way split completes the proof.

◀

▶ **Lemma 8.** *Let* $K_1 = 1/\min\{\frac{1}{d+1}, \frac{1}{8}\}$*. Then, there exist absolute constants* $c_1, c_2 > 0$ *such that the following holds for any* $\delta > 0$*:*

$$\Pr\left[|P - \mathbf{E}[P]| \geq 3\delta\right] \leq 2\exp\left\{-\frac{\delta^2}{25f_A}\right\} + 2\exp\left\{-\frac{\delta^2}{3(L-f_A)}\right\} + 2\exp\left\{-c_1\min\left(\frac{\delta^2}{c_2{}^2K_1{}^2}, \frac{\delta}{c_2K_1}\right)\right\}.$$

Proof. We characterize P by considering three separate groups of characters: the 'A' characters in S, the non-'A' characters in S, and the characters that are inserted into S (by the mutation process).

First, we observe that an 'A' in S contributes -3 to P if it was untouched by the mutation process, contributes 1 to P if it was substituted into a non-'A' character by the mutation process, and contributes 0 to P if it was deleted by the mutation process. To capture these cases, we define X_i as follows:

bioRxiv preprint doi: https://doi.org/10.1101/2025.05.14.653858; this version posted May 18, 2025. The copyright holder for this preprint (which was not certified by peer review) is the author/funder, who has granted bioRxiv a license to display the preprint in perpetuity. It is made available under aCC-BY 4.0 International license.

$$X_i = \begin{cases} -3 & \text{with probability } 1 - p_s - p_d, \\ 1 & \text{with probability } p_s, \text{ and} \\ 0 & \text{with probability } p_d; \end{cases}$$

defined for the i's such that S_i = 'A'. We recall that there are f_A such characters in S.

Next, we observe that a non-'A' character in S contributes 1 to P if it was untouched by the mutation process or substituted into another non-'A' character, contributes -3 to P if it is substituted into an 'A' character by the mutation process, and contributes 0 to P if it was deleted by the mutation process. To capture these cases, we define Y_i as follows:

$$Y_i = \begin{cases} 1 & \text{with probability } 1 - p_s/3 - p_d, \\ -3 & \text{with probability } p_s/3, \text{ and} \\ 0 & \text{with probability } p_d; \end{cases}$$

defined for the i's such that $S_i \neq$ 'A'. We recall that there are $L - f_A$ such characters in S.

And finally, we define Z_i for $i = 1$ to L as follows:

$$Z_i = l_i - 4C_i. \tag{25}$$

We can therefore characterize P in terms of X_i, Y_i, and Z_i as follows:

$$P = \sum_{i:S_i=\text{'A'}} X_i + \sum_{i:S_i\neq\text{'A'}} Y_i + \sum_{i=1}^{L} Z_i.$$

For notational simplicity, let us define the following.

$$X = \sum_{i:S_i=\text{'A'}} X_i,$$
$$Y = \sum_{i:S_i\neq\text{'A'}} Y_i, \quad \text{and}$$
$$Z = \sum_{i=1}^{L} Z_i.$$

Then, we have

$$P = X + Y + Z.$$

We first observe that the expectations of X_i, Y_i, and Z_i are easily obtained.

$$\mathbf{E}[X_i] = p_s - 3(1 - p_s - p_d) = -3 + 4p_s + 3p_d,$$
$$\mathbf{E}[Y_i] = 1 - p_s/3 - p_d - p_s = 1 - \frac{4}{3}p_s - p_d, \quad \text{and}$$
$$\mathbf{E}[Z_i] = 0.$$

bioRxiv preprint doi: https://doi.org/10.1101/2025.05.14.653858; this version posted May 18, 2025. The copyright holder for this preprint (which was not certified by peer review) is the author/funder, who has granted bioRxiv a license to display the preprint in perpetuity. It is made available under aCC-BY 4.0 International license.

Here, we have used the fact that $\mathbf{E}[l_i] = d$ and $\mathbf{E}[C_i] = d/4$. Using these three expected values, we can easily obtain the expectations of X, Y, Z, and P.

$$\mu_X = \mathbf{E}[X] = f_A(-3 + 4p_s + 3p_d),$$
$$\mu_Y = \mathbf{E}[Y] = (L - f_A)\Big(1 - \frac{4}{3}p_s - p_d\Big),$$
$$\mu_Z = \mathbf{E}[Z] = 0, \quad \text{and}$$
$$\mu_P = \mathbf{E}[P] = (L - 4f_A)\Big(1 - \frac{4}{3}p_s - p_d\Big).$$

We obtain a strong concentration of X around μ_X by applying Hoeffding's inequality shown in (20). Doing this gives us the following result, which holds for any $\delta > 0$:

$$\Pr(|X - \mu_X| \geq \delta) \leq 2e^{-\frac{\delta^2}{25 f_A}}. \tag{26}$$

A similar application of Hoeffding's inequality for Y gives us the following result, which also holds for any $\delta > 0$.

$$\Pr(|Y - \mu_Y| \geq \delta) \leq 2e^{-\frac{\delta^2}{3(L-f_A)}}. \tag{27}$$

Recall that Z is the sum of Z_i's. We prove that Z is strongly concentrated around its mean 0 by first proving that the Z_i's are sub-exponential random variables.

▶ **Lemma 15.** *For each Z_i, $i = 1$ to L, the following holds for all $t \geq 0$:*

$$\Pr[|Z_i| \geq t] \leq 2e^{-t/K_1},$$

where $\frac{1}{K_1} = \min\{\frac{1}{d+1}, \frac{1}{8}\}$.

Proof. Recall the definition of Z_i's in Equation (25). We observe that l_i follows the Geometric distribution with mean d, and C_i follows Binomial distribution, where the success probability is $1/4$, and the number of trials is l_i. Therefore, we have the following:

$$l_i \sim \text{Geom}(p),$$

and

$$C_i \sim \text{Bin}(l_i, 1/4).$$

Here, $p = 1/(d + 1)$, and the Geometric distribution supports 0, 1, 2, etc.

We also observe that $\mathbf{E}[Z_i] = d - d = 0$. We now calculate the probability that $|Z_i|$ deviates from its mean 0 by more than t as follows:

$$\begin{aligned}
\Pr[|Z_i| \geq t] &= \sum_{y \geq 0} \Pr[l_i = y] \Pr[|\text{Bin}(y, 1/4) - y/4| \geq t/4] \\
&\leq 2p \sum_{y \geq 0} (1-p)^y e^{\frac{-t^2}{8y}} \\
&\leq 2p \left(\sum_{y=0}^{\lfloor x \rfloor} (1-p)^y e^{-\frac{t^2}{8y}} + \sum_{y=\lfloor x \rfloor + 1}^{\infty} (1-p)^y e^{-\frac{t^2}{8y}} \right)
\end{aligned}$$

bioRxiv preprint doi: https://doi.org/10.1101/2025.05.14.653858; this version posted May 18, 2025. The copyright holder for this preprint (which was not certified by peer review) is the author/funder, who has granted bioRxiv a license to display the preprint in perpetuity. It is made available under aCC-BY 4.0 International license.

For $y \le t$, we have $\frac{t^2}{8y} \ge \frac{t}{8}$, so $e^{-\frac{t^2}{8y}} \le e^{-t/8}$ and we have the following.

$$\sum_{y=0}^{\lfloor t \rfloor}(1-p)^y e^{-\frac{t^2}{8y}} \le e^{-t/8}\sum_{y=0}^{\lfloor t \rfloor}(1-p)^y \le \frac{e^{-t/8}}{p}.$$

For $y > t$, we have that the following holds.

$$\sum_{y=\lfloor t \rfloor+1}^{\infty}(1-p)^y e^{-\frac{t^2}{8y}} \le \sum_{y=\lfloor t \rfloor+1}^{\infty}(1-p)^y \le \frac{(1-p)^t}{p} \le \frac{e^{-pt}}{p}.$$

Putting the bounds together, we have that the following holds.

$$\Pr[|Z_i| \ge t] \le 2\left(e^{-t/8} + e^{-pt}\right) \le 2e^{-t/K_1}$$

where $1/K_1 = \min\{p, 1/8\}$. Setting $p = 1/(d+1)$ completes the proof.

◀

Lemma 15 shows that Z_i's are sub-exponential random variables. As Z_i's are mean-zero sub-exponential random variables, we can apply Bernstein's inequality for a sum of Z_i's, which gives us the following result.

▶ **Corollary 16.** *There exist absolute constants $c_1, c_2 > 0$ such that the following holds for any $\delta > 0$*

$$\Pr\left[|Z| \ge \delta\right] \le 2\exp\left\{-c_1 \min\left(\frac{\delta^2}{{c_2}^2 {K_1}^2}, \frac{\delta}{c_2 K_1}\right)\right\} \tag{28}$$

Taking a union bound of Equations 26, 27 and 28 completes the proof. ◀

▶ **Lemma 9.** *For any $\delta \in (0,1)$:*

$$\Pr\left[\left|L' - \mathbf{E}[L']\right| \le \delta(Ld + Lp_d)\right] \ \ge \ 2\exp\left\{-\frac{L\delta^2 d^2}{2(d+1-\delta d)(d+1)}\right\} + 2\exp\left\{-\frac{Lp_d\delta^2}{3}\right\}. \tag{16}$$

Proof. We prove that L' is strongly concentrated around its expected value by proving concentration bounds for the individual pieces of Equation (22). Specifically, we prove that $\sum l_i$ and $\sum D_i$ are strongly concentrated around their respective expected values.

For the sake of notational simplicity, let us define $\mathcal{L}_{\text{ins}} = \sum_{i=1}^{L} l_i$, and $\mathcal{L}_{\text{del}} = \sum_{i=1}^{L} D_i$. Then, L' is simply

$$L' = L + \mathcal{L}_{\text{ins}} - \mathcal{L}_{\text{del}}. \tag{29}$$

We first prove a strong concentration of $\mathcal{L}_{\text{ins}}$ around its expected value, and later prove the same result for $\mathcal{L}_{\text{del}}$. We finally use these results to show that L' is strongly concentrated around its mean.

We observe that every l_i is sampled from a geometric distribution with mean $d \ge 0$. This geometric distribution has support for values 0, 1, 2, 3, etc. (opposed to supporting positive values only). Another interpretation of this Geometric distribution is that it assigns probability to the number of failures before encountering a success – where each trial is a Bernoulli trial with success probability $1/(d+1)$. We also observe that the expected value of l_i is d, and therefore, the expected value of $\mathcal{L}_{\text{ins}}$ is Ld.

We aim to determine $\Pr[\mathcal{L}_{\text{ins}} \ge (1+\delta)Ld]$. We notice that $\mathcal{L}_{\text{ins}} \ge (1+\delta)Ld$ is equivalent to observing at least $(1+\delta)Ld$ failures before observing L successes from a series of Bernoulli trials

bioRxiv preprint doi: https://doi.org/10.1101/2025.05.14.653858; this version posted May 18, 2025. The copyright holder for this preprint (which was not certified by peer review) is the author/funder, who has granted bioRxiv a license to display the preprint in perpetuity. It is made available under aCC-BY 4.0 International license.

with success probability $1/(d+1)$. Using the same argument, this is equivalent to observing at most L successes in $(1+\delta)Ld + L$ Bernoulli trials with success probability $1/(d+1)$. Let Z_i be a Bernoulli random variable with success probability $1/(d+1)$ for $i \geq 1$. Then, we have the following.

$$\Pr[\mathcal{L}_{\text{ins}} \geq (1+\delta)Ld] = \Pr[\sum_{i=1}^{(1+\delta)Ld+L} Z_i \leq L] \tag{30}$$

The expected number of success events in this collection of Bernoulli trials is $\mu = \frac{(1+\delta)Ld+L}{d+1}$. By reorganizing Equation (30), we get the following result.

$$\Pr[\mathcal{L}_{\text{ins}} \geq (1+\delta)Ld] = \Pr[\sum_{i=1}^{(1+\delta)Ld+L} Z_i \leq (1-\epsilon)\mu] \tag{31}$$

where $\epsilon = \frac{\delta d}{\delta d+d+1}$. Using Chernoff inequality for the sum of independent Bernoulli random variables from Equation (18), the probability in Equation (31) is at most $\exp\{-\epsilon^2\mu/2\}$. Using the values of μ and ϵ as defined before, we have the following result.

$$\Pr[\mathcal{L}_{\text{ins}} \geq (1+\delta)Ld] \leq \exp\{-\frac{L\delta^2 d^2}{2(d+1+\delta d)(d+1)}\} \tag{32}$$

Proceeding in an analogous manner, we can prove the probability for the other tail.

$$\Pr[\mathcal{L}_{\text{ins}} \leq (1-\delta)Ld] \leq \exp\{-\frac{L\delta^2 d^2}{2(d+1-\delta d)(d+1)}\} \tag{33}$$

Taking a union bound using the probabilities in Equation (32) and Equation (33), we have the following.

$$\Pr[|\mathcal{L}_{\text{ins}} - Ld| \leq \delta Ld] \geq 1 - 2\exp\{-\frac{L\delta^2 d^2}{2(d+1-\delta d)(d+1)}\} \tag{34}$$

We next prove that $\mathcal{L}_{\text{del}}$ is strongly concentrated around its mean. We recall that $\mathcal{L}_{\text{del}} = \sum_{i=1}^{L} D_i$, where each D_i for $i = 1$ to L is a Bernoulli trial with success probability p_d. Here, all D_i's are independent and identically distributed, and $\mathbf{E}[\mathcal{L}_{\text{del}}] = Lp_d$. Therefore, we can use the Chernoff bound in Equation (19) for $\mathcal{L}_{\text{del}}$, which gives us the following result.

$$\Pr[|\mathcal{L}_{\text{del}} - Lp_d| \leq \delta Lp_d] \quad \geq \quad 1 - 2\exp\{-\frac{Lp_d\delta^2}{3}\}. \tag{35}$$

Recall that $L' = L + \mathcal{L}_{\text{ins}} - \mathcal{L}_{\text{del}}$, as defined in Equation (29). Therefore, by using the strong concentration of $\mathcal{L}_{\text{ins}}$ and $\mathcal{L}_{\text{del}}$ around their respective expected values in Equation (34) and Equation (35), the proof is completed. ◀

▶ **Theorem 10.** *Suppose $4f_A \leq L$ and $\frac{4}{3}p_s + p_d \leq 1$. Then, for sufficiently small $\delta > 0$, there exists constants $c_1, c_2 > 0$ such that*

$$\Pr[|\hat{p}_s - p_s| \geq 4\delta] \leq 6k\exp\Big\{-\frac{\delta^2\mathbf{E}[\mathcal{N}]}{3k}\Big\}4k\exp\Big\{-\frac{\delta^2\mathbf{E}[\mathcal{D}]}{3k}\Big\}+$$
$$+2\exp\Big\{-\frac{\delta^2\mu_P^2}{225f_A}\Big\}+2\exp\Big\{-\frac{\delta^2\mu_P^2}{27(L-f_A)}\Big\}+2\exp\Big\{-c_1\min\Big(\frac{\delta^2\mu_P^2}{{c_2}^2},\frac{\delta\mu_P}{c_2}\Big)\Big\}.$$

bioRxiv preprint doi: https://doi.org/10.1101/2025.05.14.653858; this version posted May 18, 2025. The copyright holder for this preprint (which was not certified by peer review) is the author/funder, who has granted bioRxiv a license to display the preprint in perpetuity. It is made available under aCC-BY 4.0 International license.

Proof. Assume $4f_A \leq L$ and that $\frac{4}{3}p_s + p_d \leq 1$. Let $Q = k\mathcal{N} + \mathcal{D}$, $R = k(L-4f_A)\mathcal{N}$, and $T = 4k\mathcal{N} + \mathcal{D}$. Observe that

$$\hat{p}_s = -3 \cdot \frac{PQ - R}{(L - 4f_A)T};$$

our proof will transfer concentration bounds for these quantities to one for $\hat{p}_s$.

First, by Lemma 6

$$\Pr\left[|\mathcal{N} - \mathbf{E}[\mathcal{N}]| \geq \delta\mathbf{E}[\mathcal{N}]\right] \leq 2k \exp\left\{-\frac{\delta^2\mathbf{E}[\mathcal{N}]}{3k}\right\}. \tag{36}$$

So if $4f_A \leq L$, we can multiply the inequality inside the probability by $k(L - 4f_A)$ to obtain that:

$$\Pr\left[|R - \mathbf{E}[R]| \geq \delta\mathbf{E}[R]\right] \leq 2k \exp\left\{-\frac{\delta^2\mathbf{E}[\mathcal{N}]}{3k}\right\}.$$

Similarly, from Lemmas 6 and 7 and a union bound, we have that for any $\delta \geq 0$

$$\Pr\left[|Q - \mathbf{E}[Q]| \geq \delta\mathbf{E}[Q]\right] \leq 2k \exp\left\{-\frac{\delta^2\mathbf{E}[\mathcal{N}]}{3k}\right\} + 2k \exp\left\{-\frac{\delta^2\mathbf{E}[\mathcal{D}]}{3k}\right\},$$

$$\Pr\left[|T - \mathbf{E}[T]| \geq \delta\mathbf{E}[T]\right] \leq 2k \exp\left\{-\frac{\delta^2\mathbf{E}[\mathcal{N}]}{3k}\right\} + 2k \exp\left\{-\frac{\delta^2\mathbf{E}[\mathcal{D}]}{3k}\right\}, \tag{37}$$

Moreover, since under the assumptions $\mathbf{E}[P] \geq 0$, by Lemma 8, there exists constants c_1 and c_2 such that

$$\Pr\left[|P-\mathbf{E}[P]| \geq \delta\mathbf{E}[P]\right] \leq 2\exp\left\{-\frac{\delta^2\mathbf{E}[P]^2}{225 f_A}\right\} + 2\exp\left\{-\frac{\delta^2\mathbf{E}[P]^2}{27(L-f_A)}\right\} + 2\exp\left\{-c_1 \min\left(\frac{\delta^2\mathbf{E}[P]^2}{c_2{}^2}, \frac{\delta\mathbf{E}[P]}{c_2}\right)\right\}.$$

Now, let us assume that the concentration bounds above do hold and that indeed:

$$(1-\delta)\,\mathbf{E}[P] \leq P \leq (1+\delta)\,\mathbf{E}[P], \tag{38}$$

$$(1-\delta)\,\mathbf{E}[Q] \leq Q \leq (1+\delta)\,\mathbf{E}[Q], \tag{39}$$

$$(1-\delta)\,\mathbf{E}[R] \leq R \leq (1+\delta)\,\mathbf{E}[R], \tag{40}$$

$$(1-\delta)\,\mathbf{E}[T] \leq T \leq (1+\delta)\,\mathbf{E}[T]. \tag{41}$$

Then,

$$\frac{(1-\delta)^2}{1+\delta}\frac{\mathbf{E}[P]\mathbf{E}[Q]}{\mathbf{E}[T]} \leq \frac{PQ}{T} \leq \frac{(1+\delta)^2}{1-\delta}\frac{\mathbf{E}[P]\mathbf{E}[Q]}{\mathbf{E}[T]}.$$

Note that here we rely on the assumption that $\mathcal{D} > 0$, which guarantees that $T > 0$.

We observe that $\frac{(1-\delta)^2}{1+\delta} \geq 1 - 4\delta$ and $\frac{(1+\delta)^2}{1-\delta} \leq 1 + 4\delta$ for small enough δ. Then, we have the following:

$$(1-4\delta)\frac{\mathbf{E}[P]\mathbf{E}[Q]}{\mathbf{E}[T]} \leq \frac{PQ}{T} \leq (1+4\delta)\frac{\mathbf{E}[P]\mathbf{E}[Q]}{\mathbf{E}[T]} \tag{42}$$

Similarly, by dividing Equation (40) by Equation (41), and noting that that $\frac{1-\delta}{1+\delta} \geq 1 - 3\delta$, and $\frac{1+\delta}{1-\delta} \leq 1 + 3\delta$ for small enough δ, we obtain:

$$(1-3\delta)\frac{\mathbf{E}[R]}{\mathbf{E}[T]} \leq \frac{1-\delta}{1+\delta} \cdot \frac{\mathbf{E}[R]}{\mathbf{E}[T]} \leq \frac{R}{T} \leq \frac{1+\delta}{1-\delta} \cdot \frac{\mathbf{E}[R]}{\mathbf{E}[T]} \leq (1+3\delta)\frac{\mathbf{E}[R]}{\mathbf{E}[T]}.$$

bioRxiv preprint doi: https://doi.org/10.1101/2025.05.14.653858; this version posted May 18, 2025. The copyright holder for this preprint (which was not certified by peer review) is the author/funder, who has granted bioRxiv a license to display the preprint in perpetuity. It is made available under aCC-BY 4.0 International license.

Combined with Equation (42) this yields that

$$(1-4\delta)\frac{\mathbf{E}[P]\mathbf{E}[Q]}{\mathbf{E}[T]} - (1+4\delta)\frac{\mathbf{E}[R]}{\mathbf{E}[T]} \leq \frac{PQ-R}{T} \leq (1+4\delta)\frac{\mathbf{E}[P]\mathbf{E}[Q]}{\mathbf{E}[T]} - (1-4\delta)\frac{\mathbf{E}[R]}{\mathbf{E}[T]}.$$

Multiplying by $\frac{-3}{L-4f_A}$ we obtain that $p_s - 4\delta W \leq \hat{p}_s \leq p_s + 4\delta W$ with $W = 3\frac{\mathbf{E}[P]\mathbf{E}[Q]+\mathbf{E}[R]}{(L-4f_A)E[T]}$. It can be checked that $W \leq 2$, and the result then follows from a union bound.

◀

bioRxiv preprint doi: https://doi.org/10.1101/2025.05.14.653858; this version posted May 18, 2025. The copyright holder for this preprint (which was not certified by peer review) is the author/funder, who has granted bioRxiv a license to display the preprint in perpetuity. It is made available under aCC-BY 4.0 International license.

D Interesting but unused results

Herein, we describe some interesting observations and results that were uncovered in the process of finding our main results, but which ultimately were not used in any proofs or the like. These include additional statistics, concentration inequalities, and an alternate, polynomial-based mutation parameter estimation approach.

D.1 Estimating the mutation rates using a polynomial

Unlike the estimators shown in Section 4, we can estimate the mutation rates by using a different combination of observations. In this section, we assume that $\mathbf{E}[\mathcal{S}]$, $\mathbf{E}[\mathcal{D}]$, and $\mathbf{E}[\mathcal{I}]$ are all positive. This is equivalent to assuming that p_s, p_d, and d are all non-zero. We later handle the cases for $p_s = 0$, and/or $p_d = 0$, and/or $d = 0$ separately.

For the ease of algebraic manipulations, we first define the following.

$$\mathcal{S}_{\text{norm}} = \frac{\mathcal{S}}{k\,\mathcal{K}} \tag{43}$$

$$\mathcal{I}_{\text{norm}} = \frac{\mathcal{I}}{(k-1)\mathcal{K}} \tag{44}$$

$$\mathcal{D}_{\text{norm}} = \frac{\mathcal{D}}{k\,\mathcal{K}} \tag{45}$$

We first take the ratio of Equations 3 and 4 to get the following.

$$p_s = \frac{\mathbf{E}[\mathcal{S}_{\text{norm}}]}{\mathbf{E}[\mathcal{D}_{\text{norm}}]} p_d \tag{46}$$

We also take the ratio of Equations 4 and 5 to derive the following.

$$\frac{\mathbf{E}[\mathcal{D}_{\text{norm}}]}{\mathbf{E}[\mathcal{I}_{\text{norm}}]} = \frac{d+1}{d} \times \frac{p_d}{1 - \mathbf{E}[\mathcal{S}_{\text{norm}}]p_d/\mathbf{E}[\mathcal{D}_{\text{norm}}] - p_d} \tag{47}$$

By reorganizing, we get the following.

$$d + 1 = \frac{\mathbf{E}[\mathcal{D}_{\text{norm}}] - (\mathbf{E}[\mathcal{S}_{\text{norm}}] + \mathbf{E}[\mathcal{D}_{\text{norm}}])p_d}{\mathbf{E}[\mathcal{D}_{\text{norm}}] - (\mathbf{E}[\mathcal{S}_{\text{norm}}] + \mathbf{E}[\mathcal{D}_{\text{norm}}] + \mathbf{E}[\mathcal{I}_{\text{norm}}])p_d} \tag{48}$$

We next plug the values of $d + 1$ and p_s into Equation (4), and get the following polynomial after reorganizing.

$$\mathbf{E}[\mathcal{D}_{\text{norm}}]^k = p_d\Big(\mathbf{E}[\mathcal{D}_{\text{norm}}] - (\mathbf{E}[\mathcal{S}_{\text{norm}}] + \mathbf{E}[\mathcal{D}_{\text{norm}}] + \mathbf{E}[\mathcal{I}_{\text{norm}}])p_d\Big)^{k-1} \tag{49}$$

Given S and S', we can only observe $\mathcal{I}_{\text{norm}}$, $\mathcal{D}_{\text{norm}}$, and $\mathcal{I}_{\text{norm}}$. Using these observations, we can estimate p_d from Equation (49).

$$\mathcal{D}_{\text{norm}}{}^k = \hat{p}_d\Big(\mathcal{D}_{\text{norm}} - (\mathcal{S}_{\text{norm}} + \mathcal{D}_{\text{norm}} + \mathcal{I}_{\text{norm}})\hat{p}_d\Big)^{k-1} \tag{50}$$

Let $y = \mathcal{D}_{\text{norm}} - (\mathcal{S}_{\text{norm}} + \mathcal{D}_{\text{norm}} + \mathcal{I}_{\text{norm}})\hat{p}_d$. This results in the following polynomial:

bioRxiv preprint doi: https://doi.org/10.1101/2025.05.14.653858; this version posted May 18, 2025. The copyright holder for this preprint (which was not certified by peer review) is the author/funder, who has granted bioRxiv a license to display the preprint in perpetuity. It is made available under aCC-BY 4.0 International license.

$$y^k - \mathcal{D}_{\text{norm}}\, y^{k-1} + (\mathcal{S}_{\text{norm}} + \mathcal{D}_{\text{norm}} + \mathcal{I}_{\text{norm}})\mathcal{D}_{\text{norm}}{}^k = 0, \tag{51}$$

where

$$\hat{p}_d = \frac{\mathcal{D}_{\text{norm}} - y}{\mathcal{S}_{\text{norm}} + \mathcal{D}_{\text{norm}} + \mathcal{I}_{\text{norm}}}. \tag{52}$$

After solving for $\hat{p}_d$, we estimate the other two rates as follows.

$$\hat{p}_s = \frac{\mathcal{S}_{\text{norm}}}{\mathcal{D}_{\text{norm}}}\hat{p}_d \tag{53}$$

$$\hat{d} = \frac{(\mathcal{D}_{\text{norm}})\hat{p}_d}{\mathcal{D}_{\text{norm}} - (\mathcal{S}_{\text{norm}} + \mathcal{D}_{\text{norm}} + \mathcal{I}_{\text{norm}})\hat{p}_d} \tag{54}$$

We next handle the edge cases in which these equations do not work. Note that we assume that $1 - p_s - p_d > 0$. From Equation (4), we see that $p_d = 0$ if and only if $\mathbf{E}[\mathcal{D}] = 0$. Therefore, if we observe $\mathcal{D} = 0$, we estimate $\hat{p}_d = 0$. Using a similar argument, if we observe $\mathcal{S} = 0$, then we estimate $\hat{p}_s = 0$. And finally, if we observe $\mathcal{I} = 0$, then we estimate $\hat{d} = 0$.

D.2 Concentration of $f_A{}'$ around $\mathbf{E}[f_A{}']$

We next prove that $f_A{}'$ is strongly concentrated around its expected value. Recall the definition of $f_A{}'$ defined in Equation (23). For the sake of notational simplicity, we define the following.

$$\mathcal{A} = \sum_{i:S_i=\text{'A'}} A_i\,, \tag{55}$$

$$\mathcal{B} = \sum_{i:S_i\neq\text{'A'}} B_i\,, \text{ and} \tag{56}$$

$$C = \sum_{i=1}^{L} C_i, \tag{57}$$

where A_i, B_i, and C_i are the same as introduced in Section 3. Using these notations, $f_A{}'$ is simply

$$f_A{}' = \mathcal{A} + \mathcal{B} + C. \tag{58}$$

D.2.1 Strong concentration of $\mathcal{A}$ around $\mathbf{E}[\mathcal{A}]$

We note that the A_i's in Equation (55) are iid Bernoulli random variables with success probability $1 - p_s - p_d$. We also note that number of 'A' characters in S is f_A. Therefore, we have the following.

$$\mathbf{E}[\mathcal{A}] = f_A(1 - p_s - p_d) \tag{59}$$

As the $\mathcal{A}$ is a sum of iid Bernoulli random variables, we can use the Chernoff bound in Equation (19) for $\mathcal{A}$, which gives us the following result.

$$\Pr\left[\left|\mathcal{A} - \mathbf{E}[\mathcal{A}]\right| \leq \delta f_A(1 - p_s - p_d)\right] \;\geq\; 1 - 2\exp\{-\frac{\delta^2 f_A(1 - p_s - p_d)}{3}\} \tag{60}$$

bioRxiv preprint doi: https://doi.org/10.1101/2025.05.14.653858; this version posted May 18, 2025. The copyright holder for this preprint (which was not certified by peer review) is the author/funder, who has granted bioRxiv a license to display the preprint in perpetuity. It is made available under aCC-BY 4.0 International license.

D.2.2 Strong concentration of $\mathcal{B}$ around $\mathbf{E}[\mathcal{B}]$

We next prove a concentration bound for the sum of B_i's, as defined in Equation (23). We note that B_i's here are iid Bernoulli random variables, each with success probability of $p_s/3$. We also note that the number of characters in S that are not 'A' is $L - f_A$. Therefore, we have the following result.

$$\mathbf{E}[\mathcal{B}] = (L - f_A)\frac{p_s}{3} \tag{61}$$

As $\mathcal{B}$ is simply a sum of iid Bernoulli random variables, we can use the same Chernoff inequality in Equation (19) for $\mathcal{B}$, which gives us the following result.

$$\Pr\left[\left|\mathcal{B} - \mathbf{E}[\mathcal{B}]\right| \le \delta(L - f_A)\frac{p_s}{3}\right] \ge 1 - 2\exp\{-\frac{\delta^2(L - f_A)p_s}{9}\} \tag{62}$$

D.2.3 Strong concentration of C around $\mathbf{E}[C]$

We finally prove a concentration inequality for $\sum_{i=1}^{L} C_i$, as defined in Equation (23). Recall the result in Equation (34) – developed for a sum of iid geometric random variables, each with mean d. We prove a similar result for C by showing that each C_i for $i = 1$ to L is geometrically distributed with mean $d/4$.

We first recall that the probability mass function (PMF) for a geometric distribution with parameter p. If a random variable X follows a geometric distribution with probability parameter p where $0 < p \le 1$, then the PMF is as follows:

$$\Pr[X = j] = p(1 - p)^j, \tag{63}$$

where $j = 0, 1, 2, 3, \cdots$, etc. The expected value of X is given by

$$\mathbf{E}[X] = \frac{1 - p}{p}. \tag{64}$$

We can express the PMF of X using its mean. If the mean is m, then we need $p = 1/(m + 1)$, and therefore, the PMF of a geometric random variable with mean m is given by the following:

$$\Pr[X = j] = \frac{m^j}{(m + 1)^{j+1}}, \tag{65}$$

where $j = 0, 1, 2, 3, \cdots$, etc.

We next observe that C_i is the number of 'A' characters in $Q^{(i)}$, where $Q^{(i)}$ is the string inserted after S_i and before S_{i+1}. The number of characters in $Q^{(i)}$ is l_i, which follows a geometric distribution with mean d, and each character can be an 'A' with probability 1/4. Note that it immediately follows that $\mathbf{E}[C_i] = d/4$. We can therefore compute the probability that $C_i = j$ as follows:

bioRxiv preprint doi: https://doi.org/10.1101/2025.05.14.653858; this version posted May 18, 2025. The copyright holder for this preprint (which was not certified by peer review) is the author/funder, who has granted bioRxiv a license to display the preprint in perpetuity. It is made available under aCC-BY 4.0 International license.

$$\Pr[C_i = j] = \sum_{x=j}^{\infty} \Pr[C_i = j \mid l_i = x] \Pr[l_i = x] \tag{66}$$

$$= \sum_{x=j}^{\infty} \binom{x}{j} \left(\frac{1}{4}\right)^{j} \left(\frac{3}{4}\right)^{x-j} \Pr[l_i = x] \tag{67}$$

$$= \sum_{x=j}^{\infty} \binom{x}{j} \left(\frac{1}{4}\right)^{j} \left(\frac{3}{4}\right)^{x-j} \frac{d^x}{(d+1)^{x+1}} \tag{68}$$

$$= \left(\frac{1}{4}\right)^{j} \left(\frac{4}{3}\right)^{j} \frac{1}{d+1} \sum_{x=j}^{\infty} \binom{x}{j} \left(\frac{3}{4}\right)^{x} \frac{d^x}{(d+1)^{x}} \tag{69}$$

$$= \left(\frac{1}{4}\right)^{j} \left(\frac{4}{3}\right)^{j} \frac{1}{d+1} \sum_{x=j}^{\infty} \binom{x}{j} \left(\frac{3d}{4d+4}\right)^{x} \tag{70}$$

We can now use the following summation for Binoamial coefficients:

$$\sum_{x=0}^{\infty} \binom{x}{j} r^x = \frac{r^j}{(1-r)^{j+1}}, \tag{71}$$

where $|r| < 1$. In our case, $r = 3d/(4d+4)$. Our mutation process assumes $d \geq 0$, which means $4d + 4 > 3d$, and therefore, this summation can be used to simplify Equation (70).

$$\Pr[C_i = j] = \left(\frac{1}{4}\right)^{j} \left(\frac{4}{3}\right)^{j} \frac{1}{d+1} \left(\frac{3d}{4d+4}\right)^{j} \frac{1}{\left(1 - \frac{3d}{4d+4}\right)^{j+1}} \tag{72}$$

$$= \left(\frac{1}{4}\right)^{j} \left(\frac{4}{3}\right)^{j} \frac{1}{d+1} \times \frac{3^j d^j}{4^j (d+1)^j} \times \frac{4^{j+1}(d+1)^{j+1}}{(d+4)^{j+1}} \tag{73}$$

$$= \frac{4d^j}{(d+1)^{j+1}} \tag{74}$$

$$= \frac{(d/4)^j}{\left(d/4 + 1\right)^{j+1}} \tag{75}$$

This is the same form as the PMF of a geometric distribution shown in Equation (65). Since $\mathbf{E}[C_i] = d/4$, it follows that each C_i is a geometric random variable with mean $d/4$.

We can now use the result proved for sum of geometric random variables with mean d, as shown in Equation (34). Note that $C = \sum_{i=1}^{L} C_i$, which means $\mathbf{E}[C] = Ld/4$, where each C_i follows a geometric distribution with mean $d/4$. Using the result in Equation (34) gives us the following:

$$\Pr\left[\left|C - \frac{Ld}{4}\right| \leq \delta \frac{Ld}{4}\right] \geq 1 - 2\exp\left\{-\frac{L\delta^2 d^2}{2(d+4-\delta d)(d+4)}\right\}. \tag{76}$$

D.2.4 Strong concentration of $f_A{}'$ around $\mathbf{E}[f_A{}']$

We can now use the concentration bounds in Equations 60, 62, and 76 to take a union bound and prove the following.

bioRxiv preprint doi: https://doi.org/10.1101/2025.05.14.653858; this version posted May 18, 2025. The copyright holder for this preprint (which was not certified by peer review) is the author/funder, who has granted bioRxiv a license to display the preprint in perpetuity. It is made available under aCC-BY 4.0 International license.

$$\Pr\left[\left|f_A{}' - \mathbf{E}[f_A{}']\right| \leq \delta\mathbf{E}[f_A{}']\right] \leq$$
$$1 - 2\exp\left\{-\frac{\delta^2 f_A(1-p_s-p_d)}{3}\right\} - 2\exp\left\{-\frac{\delta^2(L-f_A)p_s}{9}\right\} - 2\exp\left\{-\frac{L\delta^2 d^2}{2(d+4-\delta d)(d+4)}\right\}. \tag{77}$$
